# Comparison of microbial diversity and metabolites on household and commercial *doenjang*

**DOI:** 10.1016/j.fochx.2023.101101

**Published:** 2023-12-25

**Authors:** Hee Yul Lee, Md. Azizul Haque, Du Yong Cho, Jong Bin Jeong, Ji Ho Lee, Ga Young Lee, Mu Yeun Jang, Jin Hwan Lee, Kye Man Cho

**Affiliations:** a Department of GreenBio Science and Agri-Food Bio Convergence Institute, Gyeongsang National University, Jinju 52727, Republic of Korea; b Department of Biochemistry and Molecular Biology, Hajee Mohammad Danesh Science and Technology University, Dinajpur 5200, Bangladesh; c Department of Life Resource Industry, Dong-A University, 37, Nakdong-daero 550 beon-gil, Saha-gu, Busan 49315, Republic of Korea

**Keywords:** *Doenjang*, Metagenome, Microbial diversity, Free amino acids, Volatile flavor compounds, Isoflavones

## Abstract

In this study, the microbial diversity, free amino acid (FAA), and biological activities of household *doenjang* (HDJ) from four different regions and commercial *doenjang* (CDJ) four manufacturers were analyzed. And volatile flavor compound (VFC) and isoflavone profiles were analyzed using gas chromatograph-mass spectrometer (GC-MS) and high performance liquid chromatography (HPLC), respectively. The major bacterial genus in 1HDJ and 2HDJ was *Bacillus* (97.5%), while in 3HDJ and 4HDJ, it was *Enterobacter* (47.5%) and *Pseudomonas* (80%), respectively. *Tetragenococcus* was the main bacterial genus of CDJ. The *Zygosaccharomyces* genus among yeast was comparatively high in all samples. In all samples, glutamic acid predominated among the FAAs, and the 3-methyl butanal, benzeneacetaldehyde, and diallyl disulphide were major VFCs. CDJ contained higher levels of isoflavone-glycoside and total phenolics. Except for 3HDJ and 4CDJ, the isoflavone-aglycone and total flavonoid contents were higher in HDJ. The correlation between bacterial genus and metabolited of *doenjang* showed that *Tetragenococcus* was closely related to glutamic acid, *Bacillus* was related to aglycones and ammonia, and *Pseudomonas* was highly related to isovaleric acid. While, correlation between yeast genus and metabolited of *doenjang* confirmed that *Candida*, *Hanseniaspora*, and *Saccharomyces* were related with furfural, benzeneacetaldehyde, and 3-methyl butanal, respectively. The results of this study can be utilized as basic data for the industrialization and development of *doenjang*.

## Highlights

• *Tetragenococcus* genus among bacteria was identified as predominantly only in CDJ.
• *Zygosaccharomyces* genus among yeast was identified as main in HDJ and CDJ.
• Glutamic acid, associated with a unique umami taste, was predominated in HDJ and CDJ.
• The 3-methyl butanal and benzeneacetaldehyde among VFCs were high in of HDJ and CDJ.
• The isoflavone-aglycones, TFs, and enzymatic inhibitory activity were higher in HDJ.

## 1 Introduction

In Korea, *doenjang*, one of the most representative soybean-fermented foods in Asia, is generally divided into two types: homemade and commercially manufactured. (Jeong et al., 2017, Lee et al., 2017). Due to changes in socioeconomic position, family structure, and the proportion of working women, the industrial production of *doenjang* has dramatically increased over the past three decades (Jeong et al., 2017). The commercial *doenjang* (CDJ) processing time is much shorter (1 to 3 months) than the traditional household *doenjang* (HDJ) processing time (more than 6 months). CDJ is made by combining soybean *koji* (commercial *meju*), which is a culture of *Aspergillus oryzae* and *Bacillus velezensis* on soybean (Han et al., 2023, Zhang et al., 2022), with wheat *koji* that has been infected with *A. oryzae* and *Aspergillus sojae* (Li et al., 2023) prior to a brief fermentation time (3 to 7 days). Following the addition of soybean *koji*, wheat *koji*, soybean, and salted brine (10 to 14 %), the CDJ is fermented for 30 days without aging (Jeong et al., 2017). In contrast, HDJ is prepared from 100 % soybeans that are allowed to undergo spontaneous fermentation to produce traditional *meju*. In the winter, the *meju* is fermented outside, and in the spring, it is dried in the sun. At this time, various molds, bacteria, and yeast grow on *meju*. The *meju* undergoes a brining process in which salt water (10 to 14 %) is added, followed by fermentation for 60 days. After that, this fermentation product was divided into solid and liquid parts, and generally, the solid parts were again fermented (aging for about a year) to produce the HDJ (Jeong et al., 2017). Therefore, compared to CDJ, it takes a lot of time and is difficult to industrialize due to uneven quality.

Soybeans, the primary ingredients in *doenjang*, are rich in physiologically active biomolecules, including isoflavones, polyphenols, flavonoids, soy oligosaccharides, saponins, and lecithin that may be fortified by the microbes used during fermentation (Chen et al., 2023, Lee et al., 2023). During fermentation, microbes hydrolyzed the main components, including proteins, lipids, carbohydrates, and flavonoid glycosides. At that time, several metabolites were created, including amino acids, organic acids, active metabolites, and aglycones, these metabolites increase the nutritional value of *donejang*, and decomposed soy proteins gave a distinctive flavor to *doenjang* (Zhang, Zhang, Guo, Ye, Zhao, & Huang, 2023) Previous studies, it is reported that the *Aspergillus* population was linked to sugar metabolism, *Bacillus* spp. was associated with fatty acids metabolism, and *Tetragenococcus* and *Zygosaccharomyces* were linked to amino acids metabolism (Lee et al., 2017). Therefore, the microbial of the *doenjang* plays a significant role in determining its chemical composition and quality.

Various microorganisms are not involved fermentation for CDJ. This is because the culture supported using a “starter” to speed up the fermentation process in a controlled environment (Lee, Yun, Lee, & Hong, 2022). Chun et al. (2020) reported that *Bacillus* and *Aspergillus* were the major strains involved in fermenting the traditional Korean *doenjang*. In addition to these strains, culture-independent methods showed that *Mucor*, *Leuconostoc*, *Staphylococcus*, *Penicillium*, *Cladosporium*, and *Enterococcus* play significant roles in the fermentation of *meju* (Ryu et al., 2021). Studies related to Korea's traditional *doenjang* and commercial *doenjang* include the correlation between microorganisms and metabolites in traditional soybean paste (Han et al., 2021, Kim et al., 2021) and a comparative evaluation of microorganisms and metabolites in soybean paste manufactured by two industrial manufacturers (Lee et al., 2017). It has not yet been adequately reported that comprehensively compared and analyzed the metabolites and microbial diversity of HDJ and CDJ.

Therefore, we collected four types of traditional HDJ from different cities and four types of CDJ Korea's representative industrial *doenjang* brand and analyzed the ability to inhibit α-glucosidase and pancreatic lipase, as well as their profiles of free amino acids (FAAs), isoflavones, and volatile flavor compounds (VFCs), for the industrialization and development of *doenjang*. In addition, by analyzing the bacterial and yeast diversity community that affects the quality of *doenjang*, we aim to provide basic information on microorganisms that can affect the quality of *doenjang* and apply this to the *doenjang* industry to contribute to the globalization of Korean *doenjang*.

## 2 Materials and methods

### 2.1 Doenjang samples

In this study, eight samples of *doenjang* were used. Four types of HDJ (1HDJ, 2HDJ, 3HDJ, and 4HDJ) were collected from households in four cities in South Korea. The preparation process of HDJ and CDJ were detailed in Fig. S1. These four HDJ were commonly made using soybean, *meju* (fermented soybean block), solar salt, and water and fermented and aged for over a year. Four types of CDJ (1CDJ, 2CDJ, 3CDJ, and 4CDJ) were purchased from different manufacturers in South Korea. Ingredients of 1CDJ are composed of soybean, wheat flour, salt, *meju*, alcohol, roasting soybean, and fermentation microorganisms. Ingredients of 2CDJ are composed of soybean, wheat flour, salt, wheat rice, *meju*, l-monosodium glutamate, mustard powder, and fermentation microorganisms. Ingredients of 3CDJ are composed of soybean, wheat flour, salt, wheat rice, alcohol, seasoned yeast powder, mustard powder, and fermentation microorganisms. Ingredients of 4CDJ are composed of soybean, wheat flour, salt, wheat rice, alcohol, soybean flour, soybean fermentation concentrate, l-monosodium glutamate, and fermentation microorganisms.

### 2.2 Reagents and instruments

The G-spin genomic DNA purification kits, MEGA quick-spin Total Fragment DNA Purification Kit, and Plasmid DNA Purification Kit were purchased from Intron (Intron Biotechnology, Suwon, Korea). The Luria-Bertani (LB) broth and agar media were purchased from Difco (Becton Dickinson Co., Spark, MD, USA). The enzymes α-glucosidase and pancreatic lipase, as well as the reagents of *ρ*-nitrophenyl-α-d-glucopyranoside (*ρ*-NPG) and *ρ*-nitrophenyl-butyrate (*ρ*-NPB), were bought from Sigma-Aldrich Co. (St. Louis, MO, USA) to evaluate the enzyme activities. The chemicals for measuring total phenolic (TP) and total flavonoid (TF) contents (such as the Folin-Ciocalteu’s reagent and diethylene glycol) were also purchased from Sigma-Aldrich Co. Among the twelve isoflavone standards, glycosides (daidzin, glycitin, and genistin), malonyl-glycosides (malonyldaidzin, malonylglycitin, and malonylgenistin), acetyl-glycosides (acetyldadzin, acetylglycitin, and acetyldaidzin), and aglycones (daidzein, glycitein, and genistein) were purchased from Sigma-Aldrich Co. and the LC Laboratories (Woburn, MA, USA). For the analysis, the reagents were purchased from Sigma-Aldrich and Fisher Scientific International, Inc. (Fairlawn, NJ, USA), respectively.

The pH was measured with a pH meter (MP 220 pH meter, Schwerzenbach, UK). 16S and 26S rRNA were amplified using Mastercycler pro S (Eppendorf, Hamburg, Germany). TP and TF contents and enzyme inhibition activities were evaluated by a Shimadzu UV–visible scanning spectrophotometer (UV-1800 240 V, Shimadzu CO., Kyoto, Japan). FAAs were evaluated by an L-8900 automatic analyzer (Hitachi High-Technologies Inc., Tokyo, Japan). The quantification of isoflavone was performed by high performance liquid chromatography (HPLC, Agilent 1200 system, Agilent Technologies Inc., Waldbronn, Germany) equipped with an autosampler and quaternary pump using Lichrophore 100 RP C18 (LichroCART 125–4, 5 μm, 125 mm × 4 mm, Merck KGaA, Darmstadt, Germany). The identification of VFCs was performed by a gas chromatograph-mass spectrometer (GC-MS, GC-7890A, MSD-5975C, Agilent Technologies, Santa Clara, CA, USA).

### 2.3 Measurement of pH, acidity and salinity

pH, acidity and salinity in the *doenjang* samples were analyzed using the method by Lee et al. (2023). Each of the HDJ and CDJ samples (10 g) was shaken for 30 min with 90 mL of distilled water. The supernatant was then obtained by centrifugation (13,000 rpm, 3 min), and its pH, and acidity were measured. The pH was measured with a pH meter and acidity (lactic acid, %) was determined by titrating the supernatant with 0.1 NaOH until pH was 8.2 ± 0.1. This acidity was calculated using follow Eq. (1):

(1) $$Acidity\;\left(\%\right)\;=\left(\frac{\mathrm{Vo}\mathrm{lume}\;of\;titrate\;\times \;N\;of\;titrate\;\times \;Equivalent\;weight\;for\;lactic\;acid}{\mathrm{Weight}\;of\;sample\;\times \;1000}\right)\times 100$$

Equivalent weight for lactic acid: CH_3_-CHOH-COOH, M.W. = 90.

For salinity measurements, each sample of HDJ and CDJ (10 g) was mixed with 40 mL of distilled water, and the supernatant was obtained after shaking and centrifugation, as stated above; its salinity (%) was calculated using a salt meter (PAL-03S, ATAGO, Japan).

### 2.4 Bacterial and yeast community analyses using unculture methods

Each 10 g of *doenjang* samples was mixed with 100 mL of phosphate buffered saline (pH 7.0) and shaken for 1 h. After filtering the samples through sterile gauze, the liquid supernatant was centrifuged at 13,000 rpm for 5 min at 4 °C. The G-spin genomic DNA purification kit was then used to isolate total DNA from pellet. Throughout the process, these kits were meticulously maintained following the manufacturer’s recommendations. After that, the extracted DNA was used as a template for polymerase chain reaction (PCR) to amplify the 16S rRNA and 26S rRNA genes. The bacterial-specific PCR primer 5′-CGG AGA GTT TGA TCC TGG-3′(1BF/18 mer, forward primer) and 5′-TAC GGC TAC CTT GTT ACG AC-3′ (1BR/20 mer, reverse primer) were used to amplify (40 cycles) 16S rRNA gene fragments (Cho et al., 2009a) and the yeast-specific PCR primers 5′-ACC CGC TGA AYT TAA GCA TAT-3′(3YF/21 mer, forward primer) and 5′-CTC CTT GGT CGT GTT TCA AGA CGG-3′(3YR/25 mer, reverse primer) were used to amplify (30 cycles) 26S rRNA gene fragments (Cho et al., 2009b). The 16S rRNA and 26S rRNA amplification were performed following methods described by Cho et al. (2009a) and Cho et al. (2009b), respectively. The PCR condition was followed by: initial denaturation at 95 ℃ for 5 min, 16S rRNA gene was followed by 40 cycles of denaturation at 94 ℃ for 30 *sec*, annealing at 49 ℃ for 30 *sec*, extension at 72 ℃ for 90 *sec*, and 26S rRNA gene was followed by 30 cycles of denaturation at 94 ℃ for 30 *sec*, annealing at 48 ℃ for 30 *sec*, extension at 72 ℃ for 60 *sec* after final extension at 95 ℃ for 5 min, the reaction was terminated by lowering the temperature to 4 ℃. The anticipated 16S rRNA and 26S rRNA PCR products of approximately 1.5 kb and 0.6 kb, respectively, were confirmed by electrophoresis on a 1 % agarose gel. After amplification, purification using MEGA quick-spin Total Fragment DNA Purification Kit (iNtRON Biotechnology Inc.) was performed. The purified genes were cloned in the pGEM-T easy vector (Promega, Madison, WI, USA) following the manufacturer's instructions and then transformed into *E. coli* DH5α competent cells (HIT competent cells T-DH5α, Real Biotech Corporation, Korea). Recombinant clones were picked randomly, and recombinant plasmids were extracted using the Plasmid DNA Purification Kit (iNtRON Biotechnology Inc.,). Nucleotide sequencing was analyzed by Genotech Corporation. The assembly of the nucleotide sequences was performed with the DNAMAN analysis system (Lynnon Biosoft, Quebec, Canada). All of the reference sequences were obtained from the National Center for Biotechnology Information (NCBI). The 16S and 26S rRNA sequence identity searches, sequences alignment, and the phylogenetic analysis were performed following the Cho et al. (2009a) method.

### 2.5 Measurement of FAA contents

The FAAs present in the *doenjang* samples were subjected to analysis utilizing the methodology delineated by Hwang et al. (2021). 5 mL of distilled water was used to homogenize and hydrolyze 1 g of the sample at 60 °C for 1 h using a heating block (HB-48P, Daihan Scientific, Korea). 1 mL of 10 % 5-sulfosalicylic acid dihydrate was added after the mixture had been vortexed, and kept at 4 °C for 2 h. The liquid was then centrifuged for 3 min at 13,000 rpm after being filtered with a syringe filter. Using a rotary vacuum condenser, the filtrate was concentrated under reduced pressure at 50 °C, and then it was dissolved in 2 mL of lithium buffer (pH 2.2). The mixture was then quantitatively analyzed by an auto amino acid analyzer and filtered using a 0.45 μm membrane filter (Whatman, Maidstone, UK).

### 2.6 Analysis of VFCs

The confirmation of the VFCs in the *doenjang* samples was carried out by utilizing a modified version of the method previously reported by Cho et al. (2022). Using high vacuum sublimation, VFCs from *doenjang* samples were extracted using solid-phase microextraction (SPME) fibers (DVB/CAR/PDMS, 50/30 µM, Supelco-57329-U). A 20 mL headspace vial was filled precisely with 5 mL of the *doenjang* sample supernatant, sealed with an aluminium cap, and then adsorbed onto a 100 m polydimethylsiloxane (PDMS) fiber for 8 min at 100 rpm at 100 °C. Then, using an HP-5MS (30 m × 0.25 mm, 0.25 m film thickness) column, a GC-MS was used to examine the VFCs. The GC oven was heated from 40 °C to 180 °C at a rate of 5 °C/min, maintained at 180 °C for 3 min, and then the temperature was increased to 280 °C. Sample injection was done in splitless mode (10:1). The flow rate of the carrier gas, helium, was maintained at 1 mL/min. The quadruple and injector were both set to 110 °C temperatures. Electron ionization at 69.9 eV produced mass spectra with a mass scanning range of 50 to 550. To figure out what each VFC was, the mass spectrum of each peak was recorded from the chromatogram and compared to the GC-MS NITS library.

### 2.7 Preparation of extracts for TP, TF, and isoflavone contents and enzyme activities

The extracts were produced using a modified version of the methods proposed by Hwang et al. (2021). The 20 mL of 50 % ethanol (EtOH) was used to extract the powdered sample (1.0 g) over the course of 12 h at room temperature in a shaking incubator. Before HPLC analysis, the crude supernatant was centrifuged at 3,000g for 15 min. It was then filtered using a syringe filter (0.45 μm, Whatman, Maidstone, UK). These extracts were used for the analysis of TP, TF, and isoflavone contents. The extracts were lyophilized to prepare a powder after being concentrated in a rotary evaporator (N-1300, SHANGHAI EYELA CO., LTD, China) at 60 °C. To assess the enzyme inhibition activities, the powders were diluted to concentrations of 0.25, 0.5, and 1 mg/mL with 50 % EtOH.

### 2.8 Measurement of isoflavone contents

Isoflavone analysis was conducted using HPLC, following the method described by Hwang et al. (2021). The mobile phase involved using water (solution A) and acetonitrile (solution B) with 0.2 % glacial acetic acid. With solvent A, the analytical parameters were set at 100 %/0 min, 90 %/15 min, 80 %/25 min, 75 %/30 min, and 65 %/45 min. The solvent flow rate was then adjusted to 1 mL/min at 30 °C, and 20 μL of each sample was then injected. The absorbance at 254 nm was measured using a diode array detector (Agilent 1200 series, Agilent Co.). We established calibration curves by integrating the peak areas of isoflavones in the HPLC chromatogram at 254 nm and drawing a linear curve according to the concentration. The isoflavone stock solution was diluted with DMSO to achieve a concentration of 1,000 μg/mL, and calibration curves were sequentially prepared at eight different concentrations, including 0.5, 1, 5, 10, 20, 40, 80, and 100 μg/mL. In this process, we confirmed a high linearity with correlation coefficients (*r^2^*) > 0.997 for each curve.

### 2.9 Measurement of TP and TF contents

The extracts were used to estimate TP and TF contents. The TP contents were calculated using the Folin-Denis method as outlined by Lee et al. (2018a), and the absorbance of the solution was measured at 750 nm. The TP contents were quantified utilizing the Eq. (2) procured from the gallic acid standard curve.

(2) $$TP=\left(Sample\;absorbance+0.0038\right)÷53.029$$

The method outlined by Lee et al., 2021, Lee et al., 2021 was used to determine the TF contents. The absorbance value was measured at 420 nm, and the TF contents was determined using the Eq. (3) derived from the rutin standard curve.

(3) $$TF=\left(Sample\;absorbance+0.0013\right)÷16.16$$

### 2.10 Evaluation of α-glucosidase and pancreatic lipase inhibition activities

The inhibitory activity of the extracts against α-glucosidase and pancreatic lipase enzymes was confirmed by slightly modifying the method previously reported by Hwang et al. (2021). The test tube was filled with 30 μL of each extract, 70 μL of either 1 U/mL α-glucosidase or of pancreatic lipase, and 50 μL of 200 mM sodium phosphate buffer (pH 6.8). Then, the mixture was pre-reacted at 37 °C for 10 min, and added to 100 μL of 10 mM *p*-NPG or *p*-NPB, dissolved in 200 mM sodium phosphate buffer (pH 6.8), and allowed to undergo reaction was reacted at 37 °C for 10 min. Finally, following the addition of 750 mL of 100 mM Na_2_CO_3_ to halt the reaction, absorbance at 420 nm was measured. The following formula was used to compute the inhibitory activity of α-glucosidase and pancreatic lipase as a percentage (%) by the following Eq. (4):

(4) $$Inhibition\;\left(\%\right)=\left[1-\left(\frac{\mathrm{experiment}\;sample\;absorbance}{\mathrm{negative}\;control\;absorbance}\right)\right]\;\times \;100\;$$

### 2.11 Statistical analysis

All data, except those related to bacterial and yeast communities, were expressed as a mean ± standard deviation. An analysis of variance (ANOVA) procedure followed by Duncan’s multiple range test was conducted using the Statistical analysis system (SAS, version 9.4, SAS Institute, Cary NC, USA) software. The Duncan's multiple range test was used to confirm the significant variances among all treatments with a significance level of *p* < 0.05. The correlation between microorganisms and the concentrations of each major metabolites was evaluated by Pearson's correlation test and visualized with Graphad Prism 8.0. Meanwhile, when generating a heatmap of the correlation between microbial diversities and FAA or VFCs, the data were prepared by selecting mainly detected or specific substances in all *doenjang* samples.

## 3 Results and discussion

### 3.1 Comparison of pH, acidity, and salinity in the HDJ and CDJ samples

**Fig. 1:**
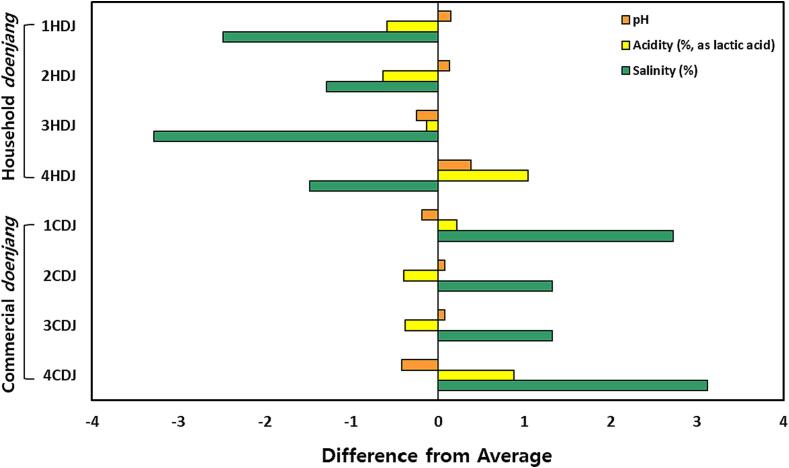
Difference of relative values from the average values of physicochemical properties of household and commercial *doenjang*.

As shown in supplementary Table S1 and Fig. 1, the pH values of HDJ and CDJ were in a weakly acidic range, varying from pH 5.14 to 5.94. Comparatively, the pH value of HDJ was higher than average values of *doenjang* samples. The acidity value was not significantly correlated with pH because, the pH value was lowest in 4CDJ (pH 5.14 and 2.87 %), while it but the acidity was highest in 4HDJ (pH 5.94 and 3.03 %), which had the highest pH. In terms of salinity, the value of HDJ was within approximately 10 %, at 8.20 to 10.20 %, and CDJ was 12.80 to 14.60 %, 2 to 4 % higher than HDJ.

HDJ samples exhibited a higher pH (5.94 to 5.31) than CDJ samples (5.64 to 5.14), consistent with previous results (Chun et al., 2020, Zhang et al., 2022). Han et al., 2021, Kim et al., 2021 and Ryu et al. (2021) reported that the pH value of *doenjang* increased during fermentation, while Tian et al. (2022) reported that it decreased. According to previous results, it is difficult to conclude that the fermentation period and pH of *doenjang* have a positive correlation. Because each report was different on pH changes during fermentation in previous research results. The difference in pH between HDJ and CDJ may be due to *koji*, which is the ingredient of CDJ, unlike HDJ. According to a study by Hong and Kim. (2020), it is reported to be the pH of rice *koji* and wheat *koji* was relatively low than soybean *koji*. Wheat koji is commonly used as a material for CDJ. On the other hand, HDJ was not used, and therefore, we think that CDJ has a relatively low pH due to this difference. Based on previous studies, the salinity concentration of *doenjang* is reported more than 11 % (11–13.61 % (traditional) and 11.27–11.60 % (commercial) (Ryu et al., 2021, Jang et al., 2021). It is generally known that traditional *doenjang* has a higher salt content than commercial *doenjang*, but the results of this study showed the opposite trend. Unlike previous results, the HDJ salinity of this study was relatively low (Fig. 1). We believe that this is a characteristic of the HDJ production method used in the experiment. It is thought that the HDJ probably had a relatively large amount of salt eluted into the liquid (soy sauce) during the divide process of the *doenjang*.

### 3.2 Comparison of microbial diversities in the HDJ and CDJ samples

#### 3.2.1 Bacterial diversity

**Fig. 2:**
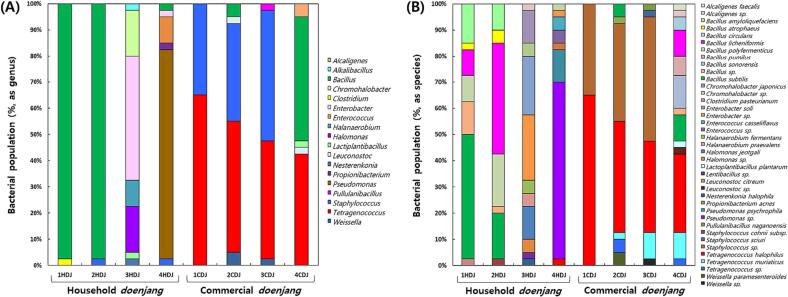

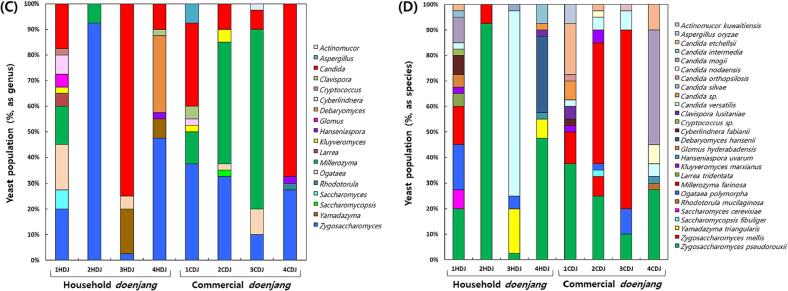
Comparisons of microbial population in household and commercial *doenjang.* (A) Bacterial population as genus, (B) Bacterial population as species, (C) Yeast population as genus, and (D) Yeast population as species.

The unculturable bacterial diversity assessd by 16S rRNA gene sequencing during the metagenomics analysis of the DNA of the HDJ and CDJ samples, is shown in Fig. 2A, and 2B, and supplementary Fig. S2 and S3. The genera of the bacterial populations in HDJ and CDJ are depicted in Fig. 2A. There were 2, 2, 7, and 6 bacterial genera in 1HDJ, 2HDJ, 3HDJ, and 4HDJ, respectively. The predominant genus in 1HDJ and 2HDJ was *Bacillus* (97.5 %), while it was *Enterobacter* (47.5 %), and *Pseudomonas* (80 %) in 3HDJ and 4HDJ, respectively. The number of bacterial genera in 1CDJ, 2CDJ, 3CDJ, and 4CDJ was confirmed to be 2, 5, 4, and 5 genus, respectively, with *Tetragenococcus* being as the main genus in proportions of 65 %, 50 %, 45 %, and 42.5 %. Additionally, *Staphylococcus* (1CDJ: 35 %, 2CDJ: 37.5 %, and 3CDJ: 50 %) was found to be the major genus in CDJ, except in 4CDJ, in which is the main genus was in *Bacillus* (47.5 %). The bacterial population, in terms of the species found in HDJ and CDJ, is depicted in Fig. 2B. Seven species were found in the 1HDJ, with *Bacillus subtilis* predominating and accounting for 47.5 % of the species (19 clones). In reality, the 2HDJ contained 7 species, with *Bacillus licheniformis* predominating and accounting for 42.5 % of the species (17 strains). The *Bacillus* genus dominated the significant majority of the bacterial populations in samples from 1HDJ and 2HDJ (97.5 %), *Clostridium* and *Staphylococcus* accounting for remaining 2.5 %. Even though 11 species, including *Alcaligenes* sp., were found in 3HDJ, *Enterobacter* sp. predominated with a 25 % presence (10 clones). In the extremely varied genus, *Enterobacter* dominated with 47.5 % of the total genus population, followed by *Halomonas* and *Chromohalobacter* with 17.5 % each. Eight species were found in 4HDJ, and a large percentage of these (67.5 %) were *Pseudomonas* sp. species (27 clones). The *Pseudomonas* genus predominated in 4HDJ, accounting for 80 % the bacterial population. Additionally, *Staphylococcus*, *Propionibacterium*, *Enterobacter*, and *Bacillus* each contributed 2.5 % of the bacterial genus, and whereas *Enterococcus* accounted for 10 %. Only two species, *Tetragenotoccus halophilus* (26 clones/65 %) and *Staphylococcus* sp., were discovered in the 1CDJ. The 2CDJ bacterial analysis led to the identification of 7 species, with the *T. halophilus* species being predominantly present (42.5 %; 17 clones). In 3CDJ, *Staphylococcus* sp. was the dominant species, accounting for 47.5 % of all species (19 clones). *T. halophilus* (14 clones/35 %) and *Tetragenococcus muriaticus* (4 clones/10 %) also predominated in the bacterial populations in 3CDJ. Of the 13 species found, *T. halophilus* predominated in 4CDJ, accounting for 30 % of the strains (12 strains), exactly as it did in 1CDJ and 2CDJ. *Bacillus sonorensis* also predominated, accounting for 12.5 % of the clones (5 clones), while *Alcaligenes faecalis*, *Alcaligenes* sp., *Lactiplantibacillus planrum, Leuconostoc* sp*.,* and *Tetragenococcus* sp*.* each contributed 2.5 % (1 clone). 1HDJ to 4HDJ included a total of 53 different bacteria, and Fig. S2 depicts the phylogenetic relationships between these. *Pseudomonas* sp. Nj-59, *B. subtilis* subsp. *subtilis* str. 168, *Bacillus polyfermenticus*, and *Enterobacter* sp. LSRC69 were the major microorganisms, among the 160 clones, accounting for 23 clones, 14 clones, 12 clones, and 10 clones, respectively. *B. polyfermenticus*, *B. subtilis* subsp. subtilis str. 168, and *Enterobacter* sp. LSRC69 all showed above 99 % similarity, whereas *Pseudomonas* sp. Nj-59 had a similarity range of 97 % to 99 %. A total of 35 bacteria were found in 1CDJ to 4CDJ, and the phylogenetic relationship between them is shown in Fig. S3. The predominant bacteria were *Staphylococcus* sp. SW80 and *T. halophilus* NBRC 12172. Of the 160 clones identified, *Staphylococcus* sp. SW80 (43 clones) showed 95 to 99 % similarity, and *T. halophilus* NBRC 12172 (55 clones) showed 94 to 100 % similarity.

The microbial composition of *doenjang* differs, depending on environmental conditions, the raw material, and salinity (Ryu et al., 2021). It has been reported that the metabolites of *doenjang* vary depending on its microbial diversity (Zhang et al., 2022). The pH has been considered one of the most important indicators of microbial growth and metabolic features during fermentation (Chun et al., 2020, Zhang et al., 2022). During the *doenjang* fermentation process, pH decreases, which is caused by acid producing-bacteria. Among acid-producing bacteria, *Staphylococcus*, *Tetragenococcus*, and *Weissella* have shown relatively high contents in CDJ, suggesting that they play an important role in the fermentation process of CDJ. Additionally, these bacteria may have affected the pH and acidity of CDJ due to their production capacity of acid during the fermentation process (Song et al., 2021). In particular, *Tetragenococcus*, a halophilic lactic acid bacteria, was reported that dominated 12–15 % salt *doenjang* samples than other lower and higher salt samples (Chun et al., 2020). Therefore, *Tetragenococcus* is believed to be dominant in CDJ with a salinity of 12.80–14.60 %. *Bacillus*, *Enterococcus*, *Staphylococcus*, and *Tetragenococcus* were dominant in *doenjang* samples (Fig. 2A), in accordance with previous results (Chun et al., 2020, Han et al., 2021, Lee et al., 2022). *Chromohalobacter* and *Halomonas* were identified only in 3HDJ and *Pseudomonas* was confirmed to be present only in 4HDJ (Fig. 2A). *Chromohalobacter* and *Halomonas*, which were halophilic bacteria, may have been derived from solar salt (Jung, Chun, & Jeon, 2015), and *Pseudomonas* in 4HDJ, might originated from *meju* (Kim, Han, Baek, Chun, & Jeon, 2022). In this way, the characteristics of the major bacteria in HDJ were confirmed to be different depending on the material used in manufacturing, whereas in CDJ, 2 to 3 genera of bacteria tended to be dominant.

#### 3.2.2 Yeast diversity

The diversity of the yeast in HDJ and CDJ determined on the basis of 26S rRNA gene sequencing is summarized and presented in Fig. 2C and 2D, and supplementary Fig. S4 and S5. The yeast population in terms of the distribution of various genus in HDJ and CDJ is depicted in Fig. 2C. The number of yeast genera in 1HDJ, 2HDJ, 3HDJ, and 4HDJ was confirmed to be 10, 2, 4, and 6, respectively. *Zygosaccharomyces* was predominated in 1HDJ, 2HDJ, and 4HDJ in proportions of 20 %, 92.5 %, and 47.5 %, respectively, while the *Candida* genus accounted for 75 % the yeast population in 3HDJ. The number of yeast genera in 1CDJ, 2CDJ, 3CDJ, and 4CDJ was confirmed to be 7, 6, 5, and 4, respectively. *Candida* and *Zygosaccharomyces* were predominant in 1CDJ (32.5 % and 37.5 %) and 4CDJ (67.5 % and 27.5 %). *Millerozyma,* the prominent genus in 2CDJ and 3CDJ, occurred with a frequency of 47.5 % and 70 %, respectively. In terms of species, 13 species were identified in 1HDJ, with *Zygosaccharomyces pseudorouxii* dominating and accounting for 20 % (8 clones) of the species. Only two species, *Millerozyma farinosa* and *Z. pseudorouxii*, were recognized in 2HDJ, accounting for 7.5 % (3 clones) and 92.5 % (37 clones) of the species, respectively. In 3HDJ, 5 species of yeast were found; *Candida versatilis* representing 72.5 % (29 clones) of the clones, was the major species, while *Yamadazyma triangularis* accounted for 17.5 % (7 clones) of the species, and other yeast represented 10 % (4 clones) of the species. 7 species were discovered in 4HDJ, including *Z. pseudorouxii* and *Debaryomyces hansenii*, accounting for 47.5 % (19 clones) and 30 % (12 clones) of the species, respectively. 7 species were discovered in 4HDJ, including *Z. pseudorouxii* and *D. hansenii*, accounting for 47.5 % (19 clones) and 30 % (12 clones) of the species, respectively. Other yeasts accounted for less than 10 % of the species. *Z. pseudorouxii* was shown to be the most prevalent species in all the samples of HDJ, except for 3HDJ (Fig. 2D). In the case of 1CDJ, 10 species were identified, with *Z. pseudorouxii* being prominent and accounting for 37.5 % (15 clones), followed by *Candida etchellsii* was accounting for 20 % (8 clones) of the species. 9 species were identified in 2CDJ; a relatively high proportion was *Z. pseudorouxii* (25 %; 10 clones) and *M. farinosa* (47.5 %; 19 clones). In 3CDJ, 5 species, including *M. farinosa*, were identified, and *M. farinosa* predominated accounting for 70 % (28 clones) of the species along with *Ogataea polynirpha* and *Z. pseudorouxii* which accounted for 10 % each. 7 species were discovered in 4CDJ, and 45 % of them belonged to *Candida mogii* (18 clones), while *Z. pseudorouxii* accounted for 27.5 % (11 clones). CDJ analysis revealed that *M. farinosa* and *Z. pseudorouxii* frequently predominated in *doenjang*, but not in all samples of CDJ. A total of 21 yeast strains were found in 1HDJ to 4HDJ, and their phylogenetic relationship is shown in Fig. S4. *C. versatilis* and *Z. pseudorouxii* ATCC 42881 were the two most prevalent yeast strains among the 21 strains, with 30 and 60 clones of the respective strains being prevalent among the 160 clones of each species as shown in Fig. S4. Both *C. versatilis* and *Z. pseudorouxii* ATCC42981 displayed 96 to 99 % and 99 to 100 % similarity, respectively (not shown). Additionally, *D. hansenii* TJY37 and *Y. triangularis* NRRLY-5714 were confirmed to constitute 12 and 10 clones among the 160 clones, respectively (not shown), and displayed 99 % similarity. Fig. S5 depicts the phylogenetic between the 22 yeast strains discovered in the CDJ samples. *M. farinosa* CBS185 and *Z. pseudorouxii* ATCC42981 were predominant among the 22 strains, accounting for 40 and 23 clones, respectively, among the 160 clones identified (not shown). Next, *C. mogii*, *Z. pseudorouxii* ABT301, and *C. etchellsii* 33Z2 constituted 18, 17, and 13 clones, respectively, and all exhibited above 99 % similarity.

According to previous studies, *Aspergillus*, *Debaryomyces*, *Millerozyma*, *Candida*, and *Zygosaccharomyces* are the predominant fungi and yeast in *doenjang*, *Candida* and *Zygosaccharomyces* were mainly present in HDJ and CDJ (Chun et al., 2020, Han et al., 2021, Lee et al., 2022). Unusually, *Aspergillus*, known as the dominant fungus in *meju* and *doenjang* and used as a starter in commercial *doenjang*, was identified only in 1CDJ. *Zygosaccharomyces* is known as a genera that improves the flavor to soy foods (Ryu et al., 2021). Also, *Candida* is known to contribute to the production of aroma components (Tanaka, Watanabe, & Mogi, 2012). Therefore, the metabolites produced by *Candida* and *Zygosaccharomyces* during the fermentation process are likely to be the main substances contributing to the flavor of HDJ and CDJ. On the other hand, *Debaryomyces* was confirmed only in 4HDJ. It is judged that *Debaryomyces* is derived from the solar salt among the ingredients used at 4HDJ, in accordance with previous result (Chun et al., 2020). Another abundant genus, *Millerozyma*, has all species identified as *M. farinosa*. *M. farinosa* is commonly found in foods such as fermented liquor, miso, and soybean paste, and has been used in food production and fermentation processes (Mallet et al., 2012). Therefore, it appears to be dominant in CDJ, and in particular, 3CDJ was present at the highest rate of 70 %. In accordance with this study, *Millerozyma*, *Candida*, and *Zygosaccharomyces* are the predominant fungi and yeast associated with fermentation in CDJ. Mostly, but not all, HDJs, *Candida* and *Zygosaccharomyces* were shown as fermenting yeast.

### 3.3 Comparison of FAAs in the HDJ and CDJ samples

**Fig. 3:**
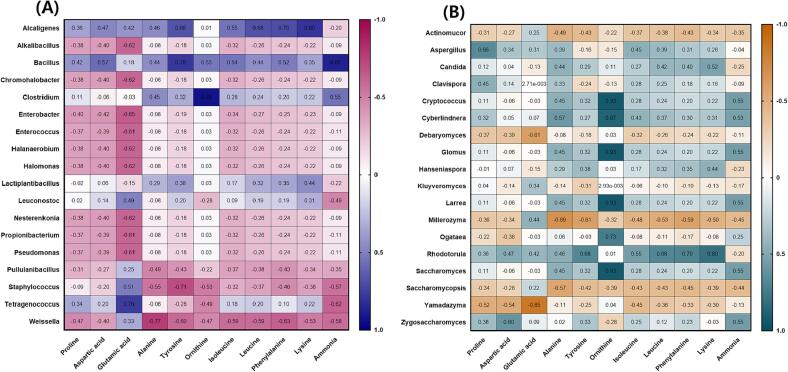
The correlation heatmap between major free amino acid contents and microorganisms in household and commercial *doenjang.* (A) Bacterial composition as genus, (B) Yeast composition as genus.

The results of the analysis of FAA contents in HDJ and CDJ samples, are shown in Fig. 3 and Table S2. There was no statistically significant difference between HDJ and CDJ in terms of overall FAAs content. Proline, aspartic acid, glutamic acid, alanine, leucine, phenylalanine, and lysine were detected as the major FAAs in HDJ and CDJ. The largest concentration of these was found to be glutamic acid. The major FAAs had no significant correlation with microorganisms (Fig. 3). The FAAs content of 1HDJ, 2HDJ, 3HDJ, and 4HDJ, respectively, was 5.37, 5.74, 2.92, and 2.93 mg/g. The FAAs concentration of 1CDJ, 2CDJ, 3CDJ, and 4CDJ was 6.78, 6.42, 0.73, and 7.22 mg/g, respectively. Among other essential amino acids, the concentration of leucine ranged from 1.48 to 2.73 mg/g in HDJ samples and from 1.07 to 3.83 mg/g in CDJ samples. Lysine showed a high concentration of 1.27 to 2.02 mg/g in HDJ samples and 0.98 to 3.00 mg/g in CDJ samples. Specifically, ammonia concentration was 0.78, 0.84, 0.49, and 0.48 mg/g in 1HDJ, 2HDJ, 3HDJ, and 4HDJ, respectively, and it was higher in HDJ than that in CDJ. Ammonia concentration had a positive correlation with *Bacillus* (*r* = 0.87, *p* = 0.05) (Fig. 3A).

Amino acids in *doenjang* are one of the most important metabolites because they contribute to the taste and flavor of *doenjang* and can be further converted into VFCs (Chun et al., 2020, Kim et al., 2020). Glutamic acid is known as the primary amino acid in *doenjang*; this study confirmed the same, except for 3CDJ (Chun et al., 2020, Han et al., 2021, Kim et al., 2021). Glutamic acid is related to the unique umami taste and provides a savory taste to the *doenjang*. In addition, in our study, aspartic acid, which contributes to the umami taste, was confirmed to be in high concentration (Gao et al., 2021), followed by proline, alanine, leucine, lysine, and phenylalanine. Likewise, other amino acids, alanine and lysine, which provide a sweet taste, were present at high levels as confirmed in 1CDJ and 4CDJ. Proline, leucine, and isoleucine, which contribute to the bitter taste, were also the principal amino acids detected in *doenjang* (Namgung et al., 2010). Tyrosine, ornithine, and lysine are the precursors of biogenic amines such as, tyramine, putrescine, and cadaverine. In the previous study, it was reported that tyramine production during the fermentation of *ganjang* might be caused by several bacteria, including *Bacillus*, *Enterococcus*, and *Tetragenococcus* (Kim et al., 2021, Han et al., 2021). Further, *Lactobacillus* and *Staphylococcus* were associated with producing putrescine and cadaverine, respectively (Kim et al., 2021, Han et al., 2021). 1HDJ, 2HDJ, and 4CDJ have a comparatively high tyrosine content, and the distribution of *Bacillus* (97.5 %, 97.5 %, and 47.5 %) is quite high; in 4CDJ the *Tetragenococcus* (42.5 %) was genus predominated. Hence, the possibility of tyramine production seems high. In the previous report, most of the amino acids were decreased in most *doenjang* after reaching their highest level at the end of fermentation, resulting in a potential decrease in the taste (Chun et al., 2020, Han et al., 2021, Kim et al., 2021, Zhang et al., 2022). Therefore, 3HDJ and 4HDJ, appear to have decreased amino acids after the fermentation period, while 2CDJ and 3CDJ, do not attain the maximum amino acid contents at the beginning of fermentation.

### 3.4 Comparison of VFCs in the HDJ and CDJ samples

**Fig. 4:**
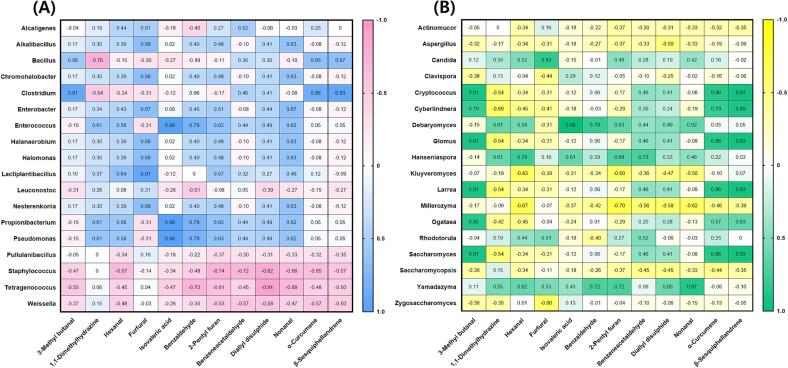
The correlation heatmap between major volatile flavor compounds and microorganisms in household and commercial *doenjang.* (A) Bacterial composition as genus, (B) Yeast composition as genus.

The results of the VFCs analysis are shown in Fig. 4 and Table S3. The 3-methyl butanal, benzeneacetaldehyde, and diallyl disulphide were detected in all samples. A total of 59 VFCs were detected in HDJ, with 3-methyl butanal, isovaleric acid, benzaldehyde, 2-pentyl furan, benzeneacetaldehyde, diallyl disulphide, nonanal, α-curcumene and β-sesquiphellandrene, being the most prevalent. 18 kinds of VFCs were detected in 1HDJ, of which 3-methyl butanal (16.38 %) accounted for a high proportion, and [3-(4-bromo-phenyl)-3-(4-trifluoromethoxy-phenyl)amino.allylidene]-4-(trifluoromethoy-phenyl)-amine (7.09 %), benzaldehyde (1.77 %), benzeneacetaldehyde (8.28 %), diallyl disulphide (3.14 %), α-curcumene (4.47 %), dihydrocurcumene (1.38 %), and β-sesquiphellandrene (2.06 %) accounted for more than 1 %. A total of 15 VFCs were detected in 2HDJ, and 3-methyl butanal (4.48 %), butanoic acid (2.44 %), and benzeneacetaldehyde (3.32 %) were predominantly present. 38 VFCs were detected in 3HDJ, among which contentrtations of tris (benzo[*b*]selenopheno) [2,3:2′,3′:2′',3′'] benzene (7.14 %), 3-methyl butanal (8.12 %), and benzeneacetaldehyde (4.32 %) were relatively high. 34 VFCs were detected in 4HDJ, and among them, benzeneacetaldehyde (8.09 %) and 3,4-dimethyl-2-hexanone (5.35 %) were present in relatively higher proportions. 32 types of VFCs were detected in CDJ. 1CDJ and 2CDJ were not detect various kinds of VFCs than in 3CDJ and 4CDJ. A total of 10 types of VFCs were detected in 1CDJ, while 9 types were detected in 2CDJ. 11 kinds of VFCs were detected in 3CDJ, with 3-methyl butanal being predominant at a concentration of 5.62 %. Finally, 28 types of VFCs were detected in 4CDJ, with benzeneacetaldehyde (8.64 %) occurring in a high proportion.

Aldehydes are produced by lipid oxidation or decomposition products during fermentation, and these metabolic activities contribute to the fruity, nutty, and roasted flavors of soybean paste. Among them, 3-methyl butanal provides a dark chocolatey and malty aroma (Jang, Yu, & Kim, 2021). It is a metabolite of leucine and is known as a major VFC in household *doenjang*, agreeing with previous studies, and has also been detected in CDJ samples (Lee, Kang, Kim, & Lim, 2015). 3-methyl butanal had a positive correlation with *Clostridium* (*p* = 0.002), *Cryptococcus* (*p* = 0.002), *Glomus* (*p* = 0.002), *Larrea* (*p* = 0.002) (Fig. 4A), and *Saccharomyces* (*p* = 0.002) (Fig. 4B). The microorganisms associated with 3-methyl butanal included more yeast than bacterial, and these are believed to contribute to improving the flavor of *doenjang*. Furfural which provides a sweet, roasted, and woody aroma was detected in one HDJ (3HDJ) and three CDJ (2CDJ to 4CDJ) samples (Lee et al., 2015, Peng et al., 2014). In a previous study, it was reported to be the predominant aldehyde compound in household *doenjang*, but our results do not agree with the previous study. Meanwhile, Furfural had a positive correlation with *Candida* (*p* = 0.011), the main yeast of HDJ and CDJ (Fig. 4B). The *Candida* genus was one of the dominant yeasts in HDJ and CDJ, except 2HDJ. The *Candida* genus is known to encourage the synthesis of aromatic compounds. Among them, *C. etchellsii* and *C. versatilis* are halophilic late-maturing yeast used in soy sauce to produce of various aromatic compounds, might have contributed to enhancing of a mature fragrance to HDJ and CDJ (Tanaka et al., 2012). *Lactiplantibacillus* (*p* = 0.002), identified only in 3HDJ, also showed a positive correlation with Furfural, which can be used as a starter to improve the flavor of CDJ in the future (Fig. 4A). Isovaleric acid, a type of carboxylic acid, known for its unpleasant odor (dirty socks odor), which is generated during the long-term storage of *doenjang*, was detected only in HDJ samples and was found to be especially high in 4HDJ with a concentration of 12.39 % (Lee et al., 2015, Peng et al., 2014). Lee et al. (2015) also reported that the isovaleric acid component comprises a substantial percentage of VFCs in traditional *doenjang*, and this study also showed similar results. Since isovaleric acid can contribute to the unpleasant odor of HDJ and reduce its quality, it is judged to be a component that needs to be managed during the fermentation process. Isovaleric acid is produced by yeast protein metabolism (Peng et al., 2014). Among yeasts, *Debaryomyces* (*p* = 0.000) showed a positive correlation with isovaleric acid (Fig. 4B). In this study, not only yeast but also bacteria such as *Pseudomonas*, *Propionibacterium*, and *Enterococcus* also showed a positive correlation with isovaleric acid. For isovaleric acid component management, the occurrence of each microorganism should be prevented. Benzaldehyde, which has the simplest structure among aromatic aldehydes, has aromatic properties similar to almonds, has a woody odor, and is easily oxidized in air, was detected in all samples except for 4CDJ (Lee et al., 2015, Peng et al., 2014). 2-pentyl furan is a metabolite found in or produced by *Saccharomyces cerevisiae*, *Aspergillus*, and bacteria (Xi et al., 2020). It is usually formed from the oxidation of linoleic acid and maillard reaction and delivers fruity, floral, buttery, green, beany, and roasted meat aromas to foods (Wang et al., 2021). 2-phentyl furan, a kind of furan derivative, was detected in all HDJ sample, was detected exceptionally in 4CDJ. It was determined to result in increased linoleic acid oxidation and maillard reaction in HDJ, due to a relatively long fermentation period. In contrast, this reaction would not have occured in CDJ, which has a relatively short fermentation period. 2-phentyl furan had a positive correlation with *Staphylococcus* (*p* = 0.042) and had a negative correlation with *Yamadazyma* (*p* = 0.036) (Fig. 4B). Benzeneacetaldehyde, aldehyde type, was confirmed to be the major VFC in HDJ and CDJ. It is generated by precursor metabolites such as amino acids, hexose, and ribose (Watanabe et al., 2015). Especially, an increase in benzeneacetaldehyde was associated with increased phenylalanine levels and contribute to flowery odor (Tamura et al., 2022, Zhang et al., 2022). Benzeneacetaldehyde had a negative correlation with *Staphylococcus* (*p* = 0.042) and a positive correlation with *Hanseniaspora* (*p* = 0.040) (Fig. 4A). Nonanal is a VFC with an unpleasant odor (fatty and citrus odor) commonly detected in HDJ, and according to the results of this study, it was identified as one of the components to be managed in traditional soybean paste along with isovaleric acid. (Jiang, Lu, Ma, Liu, & He, 2023). One common lactic acid bacteria for soybean fermentation is *T. halophilus*, which may create VFCs, such as benzaldehyde, furfural, and methyl acetate, as well as organic acids (such as lactic and acetic acid), FAAs, and different VFCs, which might impact the flavor of final products. (Tanaka et al., 2012). Inoculation of *T. halophilus* can improve the flavor of protein fermentation products (Cui, Zheng, Wu, & Zhou, 2014). In this study, *T. halophilus* showed a high distribution only in the CDJ. It seems that the manufacturer of the CDJ also added *T. halophilus* to improve the flavor. While, *Tetragenococcus* had a negative correlation with diallyl disulphide (*r* = -0.91, *p* = 0.002) (Fig. 4A). Diallyl disulphide was detected in all samples, and HDJ had a larger amount than CDJ. It contribute to alliaceous, onion, garlic, and pungent odor (Zhou et al., 2023) and was a representative active substance in garlic (Asdaq et al., 2022). HDJ would be contaminated with pathogenic microorganisms due to spontaneous fermentation, so it seems that garlic or another material similar to garlic with antibacterial activity was used to make the HDJ. Previous studies reported that esters among VFCs were identified most frequently and were present in the highest amounts in commercial and traditional China *doenjang* (Zhang et al., 2021), *miso* (Japanese *doenjang*) (Inoue et al., 2016), and Korean traditional *doenjang* (Lee et al., 2015). But, in this study, the main VFCs in HDJ and CDJ samples were the aldehydes, including 3-methyl butanal, benzaldehyde, and benzeneacetaldehyde. Aldehydes may have contributed significantly to the flavor of HCD and CDJ because they were detected in high amounts and had a low odor threshold (Inoue et al.,2016). *Clostridium*, *Cryptococcus*, *Glomus*, *Larrea*, *Saccharomyces*, and *Staphylococcus* may have been involved in accordance with this study.

### 3.5 Comparison of isoflavone, TP, and TF contents in the HDJ and CDJ samples

**Fig. 5:**
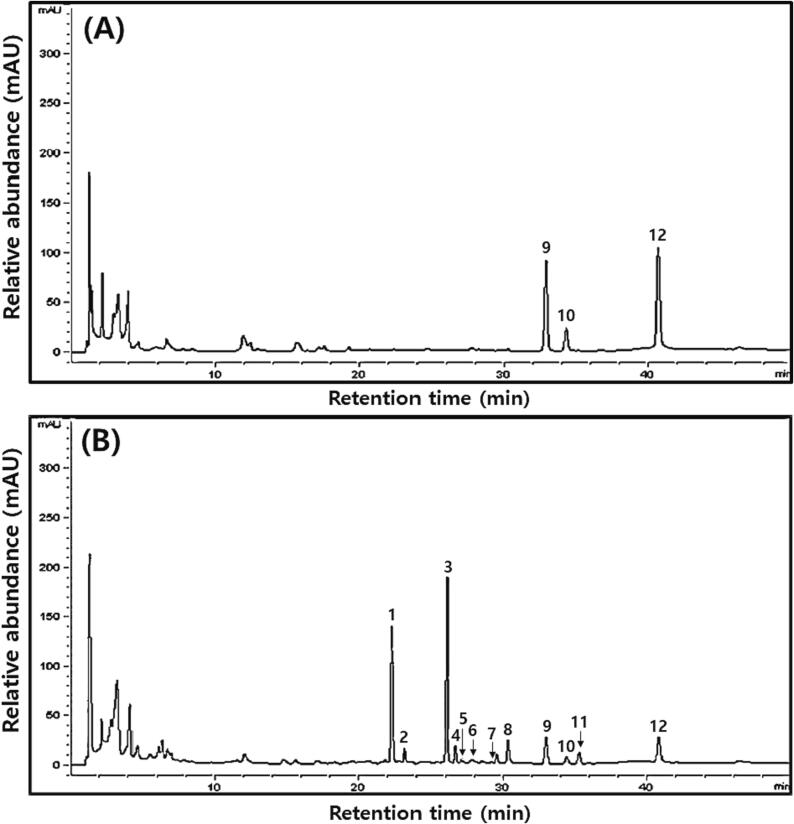
Typical HPLC chromatograms of isoflavones in household and commercial *doenjang*. (A) Household *doenjang* (3HDJ) and (B) Commercial *doenjang* (2CDJ). Chromatogram: 1. Daidzin, 2. Glycitin, 3. Genistin, 4. Malonyldaidzin, 5. Malonylglycitin, 6. Acetyldaidzin, 7. Acetylglycitin, 8. Malonylgenistin, 9. Daidzein, 10. Glycitein, 11. Acetylgenistin, and 12. Genistein.

**Fig. 6:**
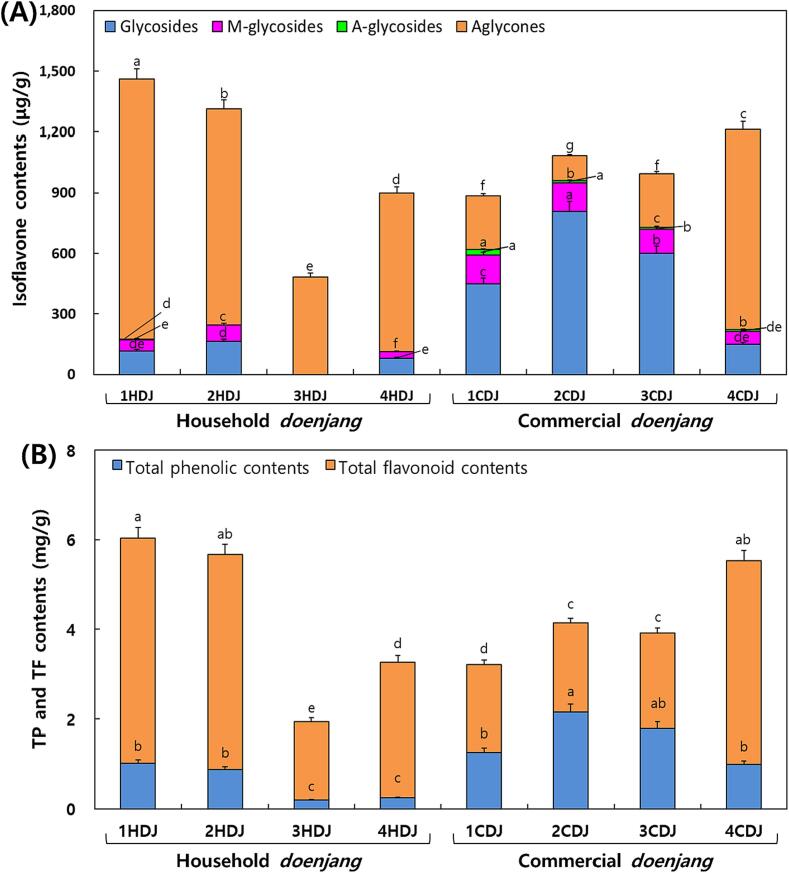
Comparison of isoflavone, total phenolic, and total flavonoid contents in household and commercial *doenjang.* (A) Isoflavone content and (B) Total phenolic Total flavonoid contents. All values are presented as the mean ± standard deviation of triplicate determination. Different letters above the bars indicate significantly different (*p* < 0.05).

**Fig. 7:**
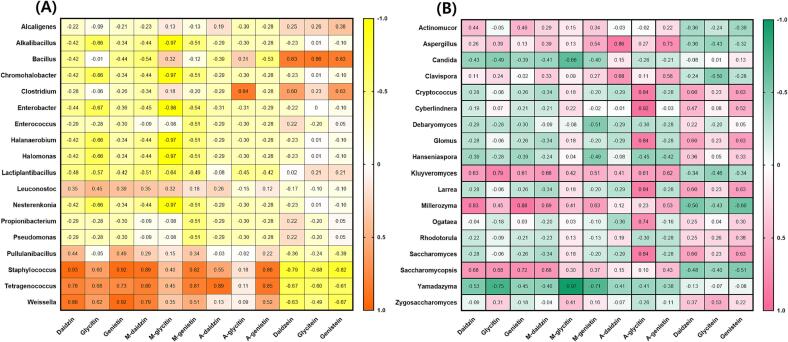
The correlation heatmap between isoflavone contents and microorganisms in household and commercial *doenjang.* (A) Bacterial composition as genus, (B) Yeast composition as genus.

Typical HPLC chromatograms of isoflavones in HDJ and CDJ samples, similar to the results shown in Fig. 5. Fig. 6A shows the changes in isoflavone glycoside and aglycone contents in the HDJ and CDJ samples. The glycoside concentrations in CDJ were 2 to 3 times higher than in HDJ. Especially, the 3HDJ never detected glycosides and just detected aglycones. Notably, the highest aglycone concentrations were found in the 1HDJ and 2HDJ samples, which were 1460.80 and 1314.22 µg/g, respectively. The 4CDJ sample showed the highest concentration of aglycones, approximately 1212.47 µg/g, and the lowest amounts of glycosides, approximately 150.47 µg/g, among the CDJ samples. Trace amounts of acetylglycosides were detected in the CDJ samples, while none were found in the HDJ samples. In particular, the 3HDJ sample showed only aglycones (such as daidzein, glycitein, and genistein) with a complete absence of glycosides (including daidzin, glycitin, genistin, malonyldaidzin, malonylglycitin, malonylgenistin, acetyldaidzin, acetylglycitin, and acetylgenistin) (Fig. 6A). Moreover, the HDJ samples showed a greater enrichment of daidzein, glycitein, and genistein compared to the CDJ samples (Fig. 6A). And daidzein, glycitein, and genistein contents had a positive correlation with *Bacillus*, and a negative correlation with *Staphylococcus* except glycitein (Fig. 7A). The differences in TP and TF contents in the HDJ and CDJ samples are presented in Fig. 6B. In the case of 1HDJ and 2HDJ samples, the concentration of TP was 1.0 mg/g, and that of TF was 5.0 mg/g. In addition, TP was detected at a concentration 0.2 mg/g in both the 3HDJ and 4HDJ samples, but TF was found at 1.8 mg/g and the TF at 3.0 mg/g in the 3HDJ and 4HDJ samples. While, TF was recorded at approximately 2.0 mg/g for the 1CDJ, 2CDJ, and 3CDJ samples, it was remarkably higher, i.e., approximately 4.7 mg/g for the 4CDJ sample. Overall, the TF contents were greater in the HDJ samples compared to the CDJ samples. The amounts of TP contents were higher in the CDJ samples than those found in the HDJ samples.

Aglycones proportation were high in HDJ, and glycosides, malonyl glucoside. On the other hands, acetyl glucoside proportation were high in CDJ, in line with earlier data (Lee et al., 2023). The TF had a large amount compare to TP in all sample, in line with previous study (Lee et al., 2021, Lee et al., 2021). However, there was a difference from the Kwon et al. (2019) reports in which soybean fermentation food including *doenjang* had higher TP contents than TF contents. TP contents were high in CDJ than HDJ, unlike previous studies (Soung et al., 2021, Lee et al., 2021). The TF contents in 1HDJ and 2HDJ were significantly higher than those that of the CDJ samples. In general, the fermentation time of HDJ was about 1 year, while the fermentation time for CDJ was 180 days. The difference in TP and TF contents was interlinked with several factors, i.e., the processing time, ingredient types, and microbial diversity (Ryu et al., 2021). Therefore, this result was judged that because the plant cell walls' glycosides are degraded to form aglycone, phenol, and flavonoid compounds by hydrolytic enzymes like β-glucosidase, decarboxylase, and esterase or microbial acids that are produced by yeast and bacteria during fermentation (Park, Park, & Chang, 2019). It was reported that fermentation of soybean foods using *Bifidobacterium longum*, *Lactiplantibacillus plantarum,* and *Levilactobacillus brevis* corresponded to a decrease in the concentration of glycoside as the fermentation process progressed (Hwang et al., 2021, Lee et al., 2022, Lee et al., 2018b). Since the fermentation period of CDJ was almost half that of the HDJ, glycoside bioconversion to aglycones was lower in CDJ, in line with the previous study, that glucoside, malonyl glucoside, and acetyl glucoside decreased and aglycone increased during the fermentation period (Kwak, Son, Chung, & Kown, 2015). In contrast, the concentrations of flavonoids were higher in CDJ (Fig. 6B).

### 3.6 Comparison of α-glucosidase and pancreatic lipase inhibitory activities in the HDJ and CDJ samples

**Fig. 8:**
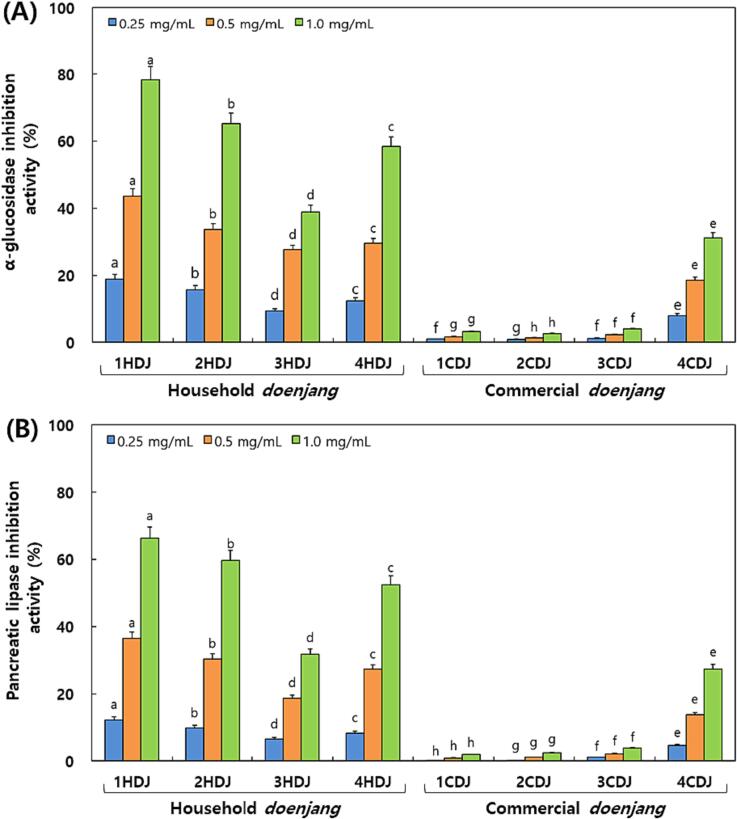
Comparisons of digestive enzymatic inhibition activities in household and commercial *doenjang.* (A) α-Glucosidase inhibition activity and (B) Pancreatic lipase inhibition activity. All values are presented as the mean ± standard of triplicate determination. Different letters above the bars indicate significantly different (*p* < 0.05).

α-glucosidase and pancreatic lipase inhibitory activities were compared between the HDJ and CDJ samples (Fig. 8). The α-glucosidase inhibitory activity was increased in response to the increased concentrations of *doenjang* extracts (0.25 to 1.0 mg/mL). The highest α-glucosidase inhibition was observed at a concentration of 64 to 78 % at 1.0 mg/mL of *doenjang* extracts (Fig. 8A). In contrast, trace levels of α-glucosidase inhibitory activity were observed in the CDJ samples 1CDJ, 2CDJ, and 3CDJ, which were about 10 to 13.5 fold lower than in the HDJ samples. However, the 4CDJ samples exhibited significantly higher α-glucosidase inhibitory activity than the other HDJ samples. According to these findings, the CDJ samples had significantly lower α-glucosidase inhibitory activity than the HDJ samples. A similar observation was found for pancreatic lipase inhibitory activity. At a concentration of 1.0 mg/mL of *doenjang* extracts, the 1HDJ, 2HDJ, 3HDJ, and 4HDJ samples showed a maximum inhibitory activity of 56 to 68 %. In contrast, the 1CDJ, 2CDJ, and 3CDJ samples demonstrated pancreatic lipase inhibitory activity below 7 %, but the 4CDJ samples showed remarkably higher activity than the other CDJ samples. None of the CDJ sample extracts have shown pancreatic lipase inhibitory activities comparable to the HDJ samples at any concentration (Fig. 8B).

The α-glucosidase and pancreatic lipase inhibitory activities were high in HDJ than CDJ (Fig. 8). The α-glucosidase inhibitory activity was 38.9–78.2 % in HDJ and 2.64–31.11 % in CDJ at 1.0 mg/mL. These values were relatively high than by Shukla et al. (2016). Previous studies have shown a positive relationship between increased concentrations of isoflavone aglycones in soybean related fermented extracts and inhibition of α-glucosidase and pancreatic lipase (Lee et al., 2018b, Lee et al., 2019, Lee et al., 2022). In this study, the contents of aglycones were higher in the HDJ extracts. Therefore, they considered that α-glucosidase and pancreatic amylase inhibitory activities were higher in the HDJ extracts (Fig. 8).

## 4 Conclusion

In this study, the differences in microbial diversity, FAA, VFC and isoflavone profiles and biological activities in HDJ and CDJ were analyzed. The major bacterial genus in 1HDJ and 2HDJ was *Bacillus* (97.5 %), while in 3HDJ and 4HDJ it was *Enterobacter* (47.5 %), and *Pseudomonas* (80 %), respectively. The main bacterial genus of CDJ was *Tetragenococcus*, occuring in proportions of 65 % (1CDJ), 50 % (2CDJ), 45 % (3CDJ), and 42.5 % (4CDJ). The *Zygosaccharomyces* genus among yeasts were found in all samples of HDJ and CDJ. The total FAA contents did not show a significant difference in HDJ and CDJ. But, glutamic acid, associated with a unique umami taste, predominated in HDJ and CDJ, together. The VFCs of 3-methyl butanal, benzeneacetaldehyde, and diallyl disulphide were detected in all samples of HDJ and CDJ. The highest concentration of 3-methyl butanal, which contributes to a dark chocolatey and malty aroma, was found in 1HDJ. The isoflavone-glycoside and TP contents in CDJ were 2 to 3 times higher than in HDJ. Notably, isoflavone-aglycone and TF contents in HDJ were higher than in CDJ, except for 3HDJ and 4CDJ. Our results provide, it is suspected that *Bacillus* is correlated with TPs, isoflavone-aglycones, *Clostridium*, *Cryptococcus*, *Glomus*, *Larrea*, and *Saccharomyces* are correlated with VFCs that improve the flavor of *doenjang*. However, no correlation has been observed with FAAs and TFs. The characteristics of microbes and components of *doenjang* according to the manufacturing methods, which can be used as basic data for the development of household and commercial *doenjang* in South Korea. In addition, microbes that show a correlation with the flavor of *doenjang* be used to new starters in the improvement of *doenjang*. However, studies with a larger amount of samples are necessary, to clarify the correlation between microbes and metabolites.

## CRediT authorship contribution statement

**Hee Yul Lee:** Methodology, Data curation, Formal analysis, Writing – original draft, Funding acquisition. **Azizul Haque:** Writing – review & editing. **Du Yong Cho:** Data curation, Visualization. **Jong Bin Jeong:** Formal analysis, Data curation. **Ji Ho Lee:** Investigation, Resources. **Ga Young Lee:** Formal analysis, Data curation. **Mu Yeun Jang:** Formal analysis, Data curation. **Jin Hwan Lee:** Conceptualization, Investigation, Validation, Writing – review & editing. **Kye Man Cho:** Conceptualization, Writing – review & editing, Project administration, Supervision, Funding acquisition.

## Declaration of competing interest

The authors declare that they have no known competing financial interests or personal relationships that could have appeared to influence the work reported in this paper.

## Data Availability

Data will be made available on request.

## References

[b0005] Asdaq S.M.B., Yasmin F., Alsalman A.J., Kamal M., Al Hawaj M.A., Alsalman K.J., Sreeharsha N. (2022). Obviation of dyslipidemia by garlic oil and its organosulfur compound, diallyl disulphide, in experimental animals. Saudi Journal of Biological Sciences.

[b0010] Chen J., Ni Y., Zhang P., Liang X., Fang S. (2023). Acidic natural deep eutectic solvents as dual solvents and catalysts for the solubilization and deglycosylation of soybean isoflavone extracts: Genistin as a model compound. Food Chemistry.

[b0020] Cho K.M., Kwon E.J., Kim S.K., Kambiranda D.M., Math R.K., Lee Y.H., Kim H. (2009). Fungal diversity in composting process of pig manure and mushroom cultural waste based on partial sequence of large subunit rRNA. Journal of Microbiology and Biotechnology.

[b0015] Cho K.M., Lee H.Y., Lee Y.M., Seo E.Y., Kim D.H., Son K.H., Lee J.H. (2022). Comparative assessment of compositional constituents and antioxidant effects in ginseng sprouts (Panax ginseng) through aging and fermentation processes. LWT.

[b0025] Cho K.M., Math R.K., Hong S.Y., Islam S.M.A., Mandanna D.K., Cho J.J., Yun H.D. (2009). Iturin produced by Bacillus pumilus HY1 from Korean soybean sauce (kanjang) inhibits growth of aflatoxin producing fungi. Food Control.

[b0030] Chun B.H., Kim K.J., Jeong S.E., Jeon C.O. (2020). The effect of salt concentrations on the fermentation of *doenjang*, a traditional Korean fermented soybean paste. Food Microbiology.

[bib261] Cui R.Y., Zheng J., Wu C.D., Zhou R.Q. (2014). Effect of different halophilic microbial fermentation patterns on the volatile compound profiles and sensory properties of soy sauce moromi. European Food Research and Technology.

[b0035] Gao B., Hu X., Li R., Zhao Y., Tu Y., Zhao Y. (2021). Screening of characteristic umami substances in preserved egg yolk based on the electronic tongue and UHPLC-MS/MS. LWT.

[b0040] Han D.M., Baek J.H., Chun B.H., Jeon C.O. (2023). Fermentative features of *Bacillus velezensis* and *Leuconostoc mesenteroides* in doenjang-meju, a Korean traditional fermented soybean brick. Food Microbiology.

[b0045] Han D.M., Chun B.H., Kim H.M., Jeon C.O. (2021). Characterization and correlation of microbial communities and metabolite and volatile compounds in *doenjang* fermentation. Food Research International.

[b0050] Hong H.H., Kim M.K. (2020). Physiochemical quality and sensory characteristics of koji made with soybean, rice, and wheat for commercial *doenjang* production. Foods.

[b0055] Hwang C.E., Kim S.C., Kim D.H., Lee H.Y., Shu H.K., Cho K.M., Lee J.H. (2021). Enhancement of isoflavone aglycone, amino acid, and CLA contents in fermented soybean yogurts using different strains: Screening of antioxidant and digestive enzyme inhibition properties. Food Chemistry.

[b0060] Inoue Y., Kato S., Saikusa M., Suzuki C., Otsubo Y., Tanaka Y., Hayase F. (2016). Analysis of the cooked aroma and odorants that contribute to umami aftertaste of soy miso (Japanese soybean paste). Food Chemistry.

[b0065] Jang H.W., Yu J.A., Kim M.K. (2021). Aroma analyses of fermented soybean paste (doenjang) using descriptive sensory analysis and μ-chamber/thermal extractor combined with thermal desorber-gas chromatography-mass spectrometry. Journal of Sensory Studies.

[b0070] Jeong D.W., Heo S., Lee B., Lee H., Jeong K., Her J.Y., Lee J.H. (2017). Effects of the predominant bacteria from meju and doenjang on the production of volatile compounds during soybean fermentation. International Journal of Food Microbiology.

[b0075] Jiang L., Lu Y., Ma Y., Liu Z., He Q. (2023). Comprehensive investigation on volatile and non-volatile metabolites in low-salt *doubanjiang* with different fermentation methods. Food chemistry.

[b0080] Jung J.Y., Chun B.H., Jeon C.O. (2015). *Chromohalobacter* is a causing agent for the production of organic acids and putrescine during fermentation of ganjang, a Korean traditional soy sauce. Journal of Food Science.

[b0085] Kim H.M., Han D.M., Baek J.H., Chun B.H., Jeon C.O. (2022). Dynamics and correlation of microbial communities and metabolic compounds in doenjang-meju, a Korean traditional soybean brick. Food Research International.

[b0090] Kim K.H., Chun B.H., Kim J.G., Jeon C.O. (2021). Identification of biogenic amine-producing microbes during fermentation of ganjang, a Korean traditional soy sauce, through metagenomic and metatranscriptomic analyses. Food Control.

[b0095] Kim S.S., Kwak H.S., Kim M.J. (2020). The effect of various salinity levels on metabolomic profiles, antioxidant capacities and sensory attributes of *doenjang*, a fermented soybean paste. Food Chemistry.

[b0100] Kwak C.S., Son D., Chung Y.S., Kwon Y.H. (2015). Antioxidant activity and anti-inflammatory activity of ethanol extract and fractions of Doenjang in LPS-stimulated RAW 264.7 macrophages. Nutrition Research and Practice.

[b0105] Kwon Y.S., Lee S., Lee S.H., Kim H.J., Lee C.H. (2019). Comparative evaluation of six traditional fermented soybean products in East Asia: A metabolomics approach. Metabolites.

[b0110] Lee H.Y., Cho D.Y., Jang K.J., Lee J.H., Jung J.G., Km M.J., Cho K.M. (2022). Changes of γ-aminobutyric acid, phytoestrogens, and biofunctional properties of the isoflavone-enriched soybean (Glycine max) leaves during solid lactic acid fermentation. Fermentation.

[b0115] Lee H.Y., Cho D.Y., Jung J.G., Kim M.J., Jeong J.B., Lee J.H., Cho K.M. (2023). Comparisons of Physicochemical Properties, Bacterial Diversities, Isoflavone Profiles and Antioxidant Activities on Household and Commercial doenjang. Molecules.

[b0120] Lee J.E., Kang S.H., Kim H.R., Lim S.I. (2015). Volatile compounds analysis of certified traditional *doenjang*. Journal of the Korean Society of Food Science and Nutrition.

[b0125] Lee J.E., Yun J.H., Lee E., Hong S.P. (2022). Untargeted Metabolomics reveals *Doenjang* metabolites affected by manufacturing process and microorganisms. Food Research International.

[b0130] Lee J.H., Hwang C.E., Cho E.J., Song Y.H., Kim S.C., Cho K.M. (2018). Improvement of nutritional components and *in vitro* antioxidative properties of soy-powder yogurt using *Lactobacillus plantarum*. Journal of Food and Drug Analysis.

[b0135] Lee J.H., Hwang C.E., Son K.S., Cho K.M. (2019). Comparisons of nutritional constituents in soybeans during solid state fermentation times and screening for their glucosidase enzymes and antioxidant properties. Food Chemistry.

[b0140] Lee J.H., Kim B., Hwang C.E., Haque M.A., Kim S.C., Lee C.S., Lee D.H. (2018). Changes in conjugated linoleic acid and isoflavone contents from fermented soymilks using *Lactobacillus plantarum* P1201 and screening for their digestive enzyme inhibition and antioxidant properties. Journal of Functional Foods.

[b0145] Lee J.H., Kim S.C., Lee H.Y., Cho D.Y., Jung J.G., Kang D.W., Cho K.M. (2021). Changes in nutritional compositions of processed mountain-cultivated ginseng sprouts (Panax ginseng) and screening for their antioxidant and anti-inflammatory properties. Journal of Functional Foods.

[b0150] Lee J.H., Seo E.Y., Lee Y.M. (2023). Comparative investigation on variations of nutritional components in whole seeds and seed coats of Korean black soybeans for different crop years and screening of their antioxidant and anti-aging properties. Food Chemistry: X.

[b0155] Lee S.H., Lee S., Lee S.H., Kim H.J., Singh D., Lee C.H. (2021). Integrated metabolomics and volatolomics for comparative evaluation of fermented soy products. Foods.

[b0160] Lee S., Lee S., Singh D., Oh J.Y., Jeon E.J., Ryu H.S., Lee C.H. (2017). Comparative evaluation of microbial diversity and metabolite profiles in *doenjang*, a fermented soybean paste, during the two different industrial manufacturing processes. Food Chemistry.

[b0165] Li J., Liu B., Feng X., Zhang M., Ding T., Zhao Y., Wang C. (2023). Comparative proteome and volatile metabolome analysis of *Aspergillus oryzae* 3.042 and *Aspergillus sojae* 3.495 during *koji* fermentation. Food Research International.

[b0170] Mallet S., Weiss S., Jacques N., Leh-Louis V., Sacerdot C., Casaregola S. (2012). Insights into the life cycle of yeasts from the CTG clade revealed by the analysis of the Millerozyma (Pichia) farinosa species complex. PLoS One.

[b0175] Namgung H.J., Park H.J., Cho I.H., Choi H.K., Kwon D.Y., Shim S.M., Kim Y.S. (2010). Metabolite profiling of *doenjang*, fermented soybean paste, during fermentation. Journal of the Science of Food and Agriculture.

[b0180] Park G., Park J.Y., Chang Y.H. (2019). Changes in flavonoid aglycone contents and antioxidant activities of citrus peel depending on enzyme treatment times. Journal of the Korean Society of Food Science and Nutrition.

[b0185] Peng X., Li X., Shi X., Guo S. (2014). Evaluation of the aroma quality of Chinese traditional soy paste during storage based on principal component analysis. Food Chemistry.

[b0190] Ryu J.A., Kim E., Yang S.M., Lee S., Yoon S.R., Jang K.S., Kim H.Y. (2021). High-throughput sequencing of the microbial community associated with the physicochemical properties of meju (dried fermented soybean) and doenjang (traditional Korean fermented soybean paste). LWT.

[b0195] Shukla S., Park J., Kim D.H., Hong S.Y., Lee J.S., Kim M. (2016). Total phenolic content, antioxidant, tyrosinase and α-glucosidase inhibitory activities of water soluble extracts of noble starter culture Doenjang, a Korean fermented soybean sauce variety. Food Control.

[b0200] Song D.H., Chun B.H., Lee S., Son S.Y., Reddy C.K., Mun H.I., Jeon C.O., Lee C.H. (2021). Comprehensive Metabolite Profiling and Microbial Communities of Doenjang (Fermented Soy Paste) and Ganjang (Fermented Soy Sauce): A Comparative Study. Foods..

[b0205] Soung S.H., Lee S., Lee S.H., Kim H.J., Lee N.R., Lee C.H. (2021). Metabolomic-based comparison of traditional and industrial Doenjang samples with antioxidative activities. Foods.

[b0210] Tamura Y., Iwatoh S., Miyaura K., Asikin Y., Kusano M. (2022). Metabolomic profiling reveals the relationship between taste-related metabolites and roasted aroma in aged pork. LWT.

[b0215] Tanaka Y., Watanabe J., Mogi Y. (2012). Monitoring of the microbial communities involved in the soy sauce manufacturing process by PCR-denaturing gradient gel electrophoresis. Food Microbiology.

[b0220] Tian Z., Ameer K., Shi Y., Yi J., Zhu J., Kang Q., Zhao C. (2022). Characterization of physicochemical properties, microbial diversity and volatile compounds of traditional fermented soybean paste in Henan province of China. Food Bioscience.

[b0225] Wang Y.H., Yang Y.Y., Li H.Q., Zhang Q.D., Xu F., Li Z.J. (2021). Characterization of aroma-active compounds in steamed breads fermented with Chinese traditional sourdough. LWT.

[b0230] Watanabe A., Kamada G., Lmanaru M., Shiba N., Yonai M., Muramoto T. (2015). Effect of aging on volatile compounds in cooked beef. Meat Science.

[b0235] Xi J., Xu D., Wu F., Jin Z., Yin Y., Xu X. (2020). The aroma compounds of Chinese steamed bread fermented with sourdough and instant dry yeast. Food Bioscience.

[b0245] Zhang K., Zhang T.T., Guo R.R., Ye Q., Zhao H.L., Huang X.H. (2023). The regulation of key flavor of traditional fermented food by microbial metabolism: A review. Food Chemistry: X.

[b0250] Zhang X., Shan T., Jia H., Guo C., Wang Z., Yue T., Yuan Y. (2022). Comparative evaluation of the effects of natural and artificial inoculation on soybean paste fermentation. LWT.

[b0255] Zhang X., Wei J., Zhao S., Jia H., Guo C., Wang Z., Gao Z., Yue T., Yuan Y. (2021). Flavor differences between commercial and traditional soybean paste. LWT.

[b0260] Zhou Q., Zheng Z., Wu Y., Zhang X., Jia Z., Zhong K., Gao H. (2023). Unraveling the core bacterial community responsible for quality and flavor improvement of the radish paocai during spontaneous fermentation. Food Bioscience.

