# Supplementary material for: Comparison of microbial diversity and metabolites on household and commercial doenjang
Source: Food Chem X. 2023 Dec 25;21:101101. doi: 10.1016/j.fochx.2023.101101 (PMC10805635; doi:10.1016/j.fochx.2023.101101)

**Supplementary data**

**Table S1. Comparisons of physicochemical properties on household and commercial *doenjang***

| **Samples** | **Index** | | |
| --- | --- | --- | --- |
|  | **pH** | **Acidity (%, as lactic acid)** | **Salinity (%)** |
| **Household *doenjang*** | | | |
| 1HDJ | 5.71±0.29a | 1.41±0.04f | 9.00±0.54d |
| 2HDJ | 5.69±0.28a | 1.36±0.05f | 10.20±0.61c |
| 3HDJ | 5.31±0.27b | 1.86±0.06d | 8.20±0.49e |
| 4HDJ | 5.94±0.30a | 3.03±0.09a | 10.00±0.60c |
| **Commercial *doenjang*** | | | |
| 1CDJ | 5.37±0.27b | 2.21±0.07c | 14.20±0.71a |
| 2CDJ | 5.64±0.25ab | 2.87±0.09b | 12.80±0.58b |
| 3CDJ | 5.64±0.34ab | 1.61±0.06e | 12.80±0.77b |
| 4CDJ | 5.14±0.26c | 1.59±0.05e | 14.60±0.88a |
| HDJ, household *doenjang*; CDJ, commercial *doenjang*; All values are presented as the mean ± standard of three independent experiments. Mean with different letters correspond to the significant difference between sample for the same index (p < 0.05). | | | |

**Table S2. Comparison of free amino acid contents in household and commercial *doenjang***

| **Contents (mg/g)** | ***Doenjang* samples** | | | | | | | |
| --- | --- | --- | --- | --- | --- | --- | --- | --- |
|  | **Household *doenjang*** | | | | **Commercial *doenjang*** | | | |
|  | **1HDJ** | **2HDJ** | **3HDJ** | **4HDJ** | **1CDJ** | **2CDJ** | **3CDJ** | **4CDJ** |
| **Non-essential amino acids** |  |  |  |  |  |  |  |  |
| *o*-Phosphoserine | 0.23±0.01a | 0.19±0.01b | 0.18±0.01b | 0.16±0.00c | 0.11±0.00d | 0.11±0.00d | 0.16±0.00c | 0.23±0.01a |
| Taurine | nd | 0.12±0.00c | nd | nd | 0.23±0.01a | 0.04±0.00d | 0.14±0.00b | nd |
| *o*-Phosphoetahnolamine | nd | 0.18±0.01a | nd | 0.01±0.00c | nd | 0.03±0.00b | nd | 0.17±0.01a |
| Proline | 1.32±0.04c | 1.57±0.05b | 0.61±0.00f | 0.63±0.02f | 2.12±0.05a | 0.67±0.02f | 0.71±0.02d | 1.69±0.05b |
| Aspartic acid | 1.70±0.05d | 3.72±0.11a | 0.66±0.02f | 0.69±0.02f | 2.95±0.08c | 1.01±0.03e | 1.05±0.03e | 3.37±0.10b |
| Serine | 1.48±0.04c | 1.37±0.04d | 0.67±0.00e | 0.67±0.01e | 1.76±0.05b | 0.52±0.02g | 0.59±0.02f | 2.00±0.06a |
| Glutamic acid | 5.37±0.16f | 5.74±0.17e | 2.92±0.09g | 2.93±0.09g | 6.78±0.18b | 6.42±0.19cd | 6.53±0.12c | 7.22±0.21a |
| Sarcosine | nd | nd | nd | nd | 0.12±0.00 | nd | nd | nd |
| Aminoadipic acid | 0.36±0.01c | 0.22±0.01f | 0.33±0.01d | 0.33±0.01d | 0.49±0.01b | 0.28±0.01e | 0.36±0.01c | 0.55±0.02a |
| Glycine | 0.78±0.02b | 0.76±0.02b | 0.48±0.01c | 0.49±0.01c | 1.04±0.03a | 0.28±0.01f | 0.37±0.01d | 1.05±0.03a |
| Alanine | 2.10±0.06a | 1.37±0.04b | 1.37±0.04b | 1.36±0.04b | 2.01±0.03a | 0.69±0.02d | 0.79±0.02c | 2.11±0.06a |
| Citrulline | 0.31±0.01d | 0.40±0.01c | 1.15±0.03a | 1.06±0.03a | nd | 0.05±0.00f | nd | 0.10±0.00e |
| α-aminobutyric acid | 0.11±0.00c | 0.09±0.00e | 0.10±0.00d | 0.10±0.00d | 0.16±0.00b | 0.09±0.00e | 0.08±0.00f | 0.19±0.01a |
| Cysteine | 0.20±0.01b | 0.10±0.00c | 0.04±0.00d | 0.04±0.00d | 0.25±0.01a | 0.04±0.00d | 0.13±0.00b | 0.23±0.01a |
| Cystathionine | 0.16±0.00b | 0.05±0.00f | 0.06±0.00e | 0.06±0.00e | 0.17±0.00a | 0.02±0.00f | 0.11±0.00d | 0.14±0.00c |
| Tyrosine | 1.43±0.04b | 1.48±0.04b | 0.76±0.01c | 0.76±0.01c | 0.78±0.02c | 0.44±0.01d | 0.42±0.00e | 1.90±0.06a |
| β-Alanine | 0.11±0.00d | 0.15±0.01c | 0.01±0.00e | 0.01±0.00e | 0.43±0.01a | 0.24±0.00b | 0.11±0.00d | 0.23±0.01b |
| β-aminoisobutyric acid | 0.38±0.01a | 0.17±0.01c | 0.01±0.00f | 0.02±0.00f | 0.18±0.01c | 0.28±0.00b | 0.16±0.00c | 0.14±0.00d |
| γ-aminobutyric acid | 0.01±0.00f | 0.13±0.00c | 0.24±0.01a | 0.24±0.00a | 0.23±0.01a | 0.09±0.00d | 0.08±0.00e | 0.14±0.00c |
| Aminoetahnol | 0.04±0.00b | nd | nd | nd | 0.04±0.00b | 0.03±0.00c | 0.03±0.00c | 0.05±0.00a |
| Hydroxylysine | 0.03±0.00b | 0.01±0.00c | 0.03±0.00b | 0.03±0.00b | 0.25±0.00a | 0.01±0.00c | 0.01±0.00c | 0.03±0.00c |
| Ornithine | 1.12±0.03a | 0.19±0.00e | 0.40±0.01b | 0.40±0.00b | 0.26±0.00c | 0.07±0.00f | 0.20±0.00d | 0.39±0.01c |
| Anserine | 0.38±0.01b | nd | 0.10±0.00f | 0.11±0.00f | 0.29±0.00c | 0.24±0.00e | 0.25±0.00d | 0.57±0.02a |
| Carnosine | 0.23±0.01b | 0.08±0.00d | 0.07±0.00e | 0.06±0.00e | 0.04±0.00g | nd | 0.09±0.00c | 0.41±0.00a |
| Arginine | 1.08±0.03e | 1.30±0.04c | 0.12±0.00f | 0.12±0.00f | 2.67±0.07b | 1.13±0.03d | 1.01±0.21de | 3.38±0.10a |
| Total | 18.93 | 19.39 | 10.31 | 10.28 | 23.37 | 12.78 | 13.38 | 26.29 |
| **Essential amino acids** |  |  |  |  |  |  |  |  |
| Threonine | 1.29±0.04b | 1.12±0.03c | 0.51±0.02d | 0.51±0.02d | 1.31±0.04b | 0.42±0.01e | 0.43±0.01e | 1.56±0.04a |
| Valine | 1.63±0.05b | 1.55±0.05c | 0.74±0.02d | 0.73±0.02d | 1.63±0.05b | 0.53±0.02f | 0.59±0.02e | 2.05±0.05a |
| Methionine | 0.27±0.01b | 0.26±0.01b | 0.16±0.00c | 0.16±0.00c | 0.56±0.02a | 0.15±0.00d | 0.27±0.01b | 0.57±0.01a |
| Isoleucine | 1.67±0.05c | 1.49±0.04d | 0.69±0.02e | 0.69±0.02e | 1.95±0.06b | 0.51±0.02g | 0.61±0.02f | 2.11±0.06a |
| Leucine | 2.73±0.08c | 2.19±0.07d | 1.49±0.03e | 1.48±0.04e | 3.12±0.08b | 1.07±0.03g | 1.18±0.04f | 3.83±0.10a |
| Phenylalanine | 1.87±0.06c | 1.79±0.05d | 1.15±0.04e | 1.15±0.03e | 2.06±0.04b | 0.79±0.02g | 0.83±0.02f | 2.70±0.08a |
| Lysine | 2.02±0.36b | 1.41±0.04c | 1.27±0.04d | 1.27±0.04d | 2.11±0.05b | 0.98±0.03f | 1.07±0.03e | 3.00±0.09a |
| Histamine | 0.52±0.02b | 0.41±0.01c | 0.30±0.00de | 0.29±0.01e | 0.32±0.01d | 0.14±0.00f | 0.08±0.00g | 0.75±0.01a |
| Total | 12.00 | 10.22 | 6.31 | 6.28 | 13.06 | 4.59 | 5.06 | 16.57 |
| **Total amino acids** | 30.93 | 29.61 | 16.62 | 16.56 | 36.43 | 17.37 | 18.44 | 42.86 |
| Ammonia | 0.78±0.02b | 0.84±0.03a | 0.49±0.01c | 0.48±0.01c | 0.51±0.02c | 0.33±0.01f | 0.37±0.01e | 0.44±0.01d |
| All values are presented as the mean ± standard deviation of triplicate determination. Means with different letters within a row are significantly different between sample for the same index (p < 0.05); nd: not detected. | | | | | | | | |

**Table S3. Comparison of volatile flavor compounds in household and commercial *doenjang***

| **No** | **Volatile compounds (%, as area)** | **R.T.**  **(min)** | ***Doenjang* samples** | | | | | | | |
| --- | --- | --- | --- | --- | --- | --- | --- | --- | --- | --- |
|  |  |  | **Household *doenjang*** | | | | **Commercial *doenjang*** | | | |
|  |  |  | **1HDJ** | **2HDJ** | **3HDJ** | **4HDJ** | **1CDJ** | **2CDJ** | **3CDJ** | **4CDJ** |
| 1 | Imipramine | 1.672 | nd | nd | nd | nd | nd | 3.46±0.06 | nd | nd |
| 2 | 2-Methyl-2-butanamine | 1.689 | nd | nd | nd | nd | nd | nd | 4.10±0.07 | nd |
| 3 | 3-Hexanamine | 1.695 | nd | 2.45±0.04 | nd | nd | nd | nd | nd | nd |
| 4 | Silacyclopentane | 1.695 | nd | nd | nd | nd | 2.09±0.05 | nd | nd | nd |
| 5 | [3-(4-Bromo-phenyl)-3-(4-trifluoromethoxy-phenyl)amino.allylidene]-4-(trifluoromethoxy-phenyl)-amine | 1.764 | 7.09±0.15 | nd | nd | nd | nd | nd | nd | nd |
| 6 | 3,4-Dimethyl-2-hexanone | 1.775 | nd | nd | nd | 5.35±0.10 | nd | nd | nd | nd |
| 7 | 6-Dimethylamino-4,4-diphenyl-3-heptanone | 1.777 | nd | nd | 3.34±0.10 | nd | nd | nd | nd | nd |
| 8 | Tris(benzo[b]selenopheno)[2,3:2',3':2'',3'']benzene | 1.781 | nd | nd | 7.14±0.20 | nd | nd | nd | nd | nd |
| 9 | 3-Methyl butanal | 2.084 | 16.38±0.41a | 4.48±0.11d | 8.12±0.20b | 4.54±0.06d | 2.55±0.05e | 2.11±0.05f | 5.62±0.14c | 5.72±0.14c |
| 10 | Symclosene | 2.565 | 0.25±0.00 | nd | nd | nd | nd | nd | nd | nd |
| 11 | Cinnamic acid p-(trimethylsiloxy)-methyl ester | 2.588 | nd | nd | nd | 0.58 | nd | nd | nd | nd |
| 12 | *trans*-2-Dodecene | 2.599 | nd | 1.16±0.03 | nd | nd | nd | nd | nd | nd |
| 13 | 2-methyl Hexanoic Acid | 2.691 | nd | nd | 1.34±0.06 | nd | nd | nd | nd | nd |
| 14 | Carbonyl sulfide | 2.822 | nd | 1.34±0.02 | nd | nd | nd | nd | nd | nd |
| 15 | 1,1-Dimethylhydrazine | 2.828 | nd | nd | 2.87±0.07b | 3.92±0.10a | 1.26±0.03f | 2.37±0.06d | 1.85±0.05e | 2.48±0.06c |
| 16 | Diethylhydroxylamine | 3.120 | nd | nd | nd | nd | 0.80±0.00 | nd | 0.76±0.00 | nd |
| 17 | Hexanal | 3.189 | nd | 1.26±0.02d | 3.40±0.06c | 4.28±0.08a | nd | nd | nd | 3.63±0.05b |
| 18 | Propanoic acid | 3.223 | nd | nd | nd | nd | nd | 0.58±0.00 | nd | nd |
| 19 | Hexanoic acid | 3.395 | 3.00 | nd | 2.63±0.05 | nd | nd | nd | nd | nd |
| 20 | Furfural | 3.647 | nd | nd | 3.71±0.07a | nd | nd | 0.76±0.02d | 1.75±0.04c | 3.09±0.07b |
| 21 | Butanoic acid | 3.755 | nd | 2.44±0.04 | nd | nd | 1.50±0.02 | 0.69±0.01 | nd | nd |
| 22 | Diallyl sulfide | 4.036 | nd | nd | nd | nd | nd | nd | 1.00±0.01 | 0.81±.01 |
| 23 | Isovaleric acid | 4.339 | 0.63±0.01c | 0.31±0.00d | 2.18±0.04b | 12.39±0.41a | nd | nd | nd | nd |
| 24 | Heptanal | 4.739 | nd | nd | 0.67±0.01 | nd | nd | nd | nd | 0.49±0.00 |
| 25 | Hexanedial | 4.756 | 0.24±0.00 | nd | nd | nd | nd | nd | nd | nd |
| 26 | Dimethyl sulfone | 5.672 | nd | nd | nd | 0.13 | nd | nd | nd | nd |
| 27 | Benzaldehyde | 5.998 | 1.77±0.03c | 1.18±0.02d | 3.10±0.04b | 4.58±0.05a | 0.52±0.01g | 0.54±0.01f | 0.70±0.01e | nd |
| 28 | 2-Ethylcyclohexanol | 6.359 | nd | nd | nd | nd | nd | nd | nd | 0.59±0.00 |
| 29 | Bicyclo[2.2.1]heptan-2-ol | 6.370 | nd | nd | 0.62±0.01b | 0.71±0.02a | nd | nd | nd | nd |
| 30 | 1-ethenyl-Cyclododecanol | 6.382 | 0.26±0.01 | nd | nd | nd | nd | nd | nd | nd |
| 31 | 2-Pentyl furan | 6.662 | 0.61±0.01e | 0.84±±0.02d | 2.57±0.05b | 3.02±0.04a | nd | nd | nd | 1.94±0.02c |
| 32 | E-4-Tridecen-1-yl acetate | 6.914 | nd | nd | nd | 0.78±0.01 | nd | nd | nd | nd |
| 33 | Octanal | 6.937 | nd | nd | 0.65±0.01 | nd | nd | nd | nd | 0.45±0.01 |
| 34 | Methyl dec-4-ynoate | 6.948 | 0.37±0.01 | nd | nd | nd | nd | nd | nd | nd |
| 35 | 3-Ethyl-1,4-hexadiene | 7.148 | nd | nd | 0.17±0.01 | nd | nd | nd | nd | 0.21±0.00 |
| 36 | 3-Ethyl-2-methyl-1,3-hexadiene | 7.692 | nd | nd | nd | 0.14±0.00 | nd | nd | nd | 0.20±0.00 |
| 37 | exo-2-Bromonorbornane | 7.698 | nd | nd | 0.16±0.01 | nd | nd | nd | nd | nd |
| 38 | Benzeneacetaldehyde | 7.978 | 8.28±0.18b | 3.32±0.09e | 4.32±0.08d | 8.09±0.15c | 2.70±0.06g | 1.83±0.03h | 2.91±0.04f | 8.64±0.32a |
| 39 | *trans*-2-Octenal | 8.327 | nd | nd | 0.29±0.01b | 0.28±0.00b | nd | nd | nd | 0.59±0.01a |
| 40 | 1-Octanol | 8.642 | nd | nd | 0.21±0.01b | 0.24±0.00a | nd | nd | nd | nd |
| 41 | Diallyl disulphide | 8.894 | 3.14±0.06b | 2.01±0.02b | 3.13±0.05b | 3.36±0.04a | 0.56±0.01e | 0.70±0.01d | 1.11±0.03c | 1.81±0.04b |
| 42 | Tetramethylpyrazine | 9.139 | 0.38±0.01b | nd | 0.34±0.00c | 0.45±0.01a | nd | nd | nd | nd |
| 43 | Guaiacol | 9.162 | nd | nd | nd | nd | nd | nd | nd | 0.71±0.00 |
| 44 | Nonanal | 9.529 | 0.69±0.02c | 0.50±0.01d | 2.63±0.05a | 2.61±0.05a | nd | nd | nd | 0.84±0.01b |
| 45 | Benzyl nitrile | 10.495 | nd | nd | nd | 0.10±0.00 | nd | nd | nd | nd |
| 46 | Decamethylcyclopentasiloxane | 10.896 | nd | nd | 0.26±0.01b | nd | nd | nd | nd | 0.55±0.00a |
| 47 | *trans*-2-Nonenal | 11.033 | nd | nd | nd | 0.49±0.00 | nd | nd | nd | nd |
| 48 | 2-Ethyl-phenol | 11.171 | nd | nd | 0.55±0.01 | nd | nd | nd | nd | nd |
| 49 | Isopropyl octanoate | 11.337 | nd | nd | nd | 0.17±0.00 | nd | nd | nd | nd |
| 50 | Ethyl caprylate | 12.041 | nd | nd | 0.14±0.00b | nd | nd | nd | nd | 0.19±0.00a |
| 51 | *trans*-2-Dodecen-1-ol trifluoroacetate | 12.275 | nd | nd | nd | 0.10±0.00 | nd | nd | nd | nd |
| 52 | Ethyl phenylacetate | 13.357 | nd | nd | 0.44±0.00b | 1.08±0.03a | nd | nd | nd | nd |
| 53 | 2-Cyclohexen-1-ol | 13.792 | nd | nd | 0.36±0.00 | nd | nd | nd | nd | 0.36±0.00 |
| 54 | *trans*-2-Decenal | 13.797 | nd | nd | nd | 0.11±0.00 | nd | nd | nd | nd |
| 55 | *p*-Ethylguaiacol | 14.278 | nd | nd | 0.16±0.00 | nd | nd | nd | nd | nd |
| 56 | 2-Octylfuran | 14.650 | nd | nd | 0.41±0.01 | nd | nd | nd | nd | nd |
| 57 | Indole | 14.656 | nd | nd | nd | 0.18±0.00 | nd | nd | nd | nd |
| 58 | (E,E)-2,4-Decadienal | 15.245 | 0.30±0.00d | nd | 1.24±0.02b | 0.49±0.01c | nd | nd | nd | 3.21±0.04a |
| 59 | Dodecamethylcyclohexasiloxane | 15.560 | nd | nd | 0.25±0.00b | nd | nd | nd | nd | 0.46±0.01a |
| 60 | E-15-Heptadecenal | 16.469 | nd | nd | 0.31±0.00 | nd | nd | nd | nd | nd |
| 61 | (2E)-2-Tridecenal | 16.471 | nd | nd | nd | 0.12±0.00 | nd | nd | nd | nd |
| 62 | 2-Cyclohexen-1-ol | 16.475 | nd | nd | nd | nd | nd | nd | nd | 0.38±0.00 |
| 63 | 3-Pentyl-1-cyclohexene | 16.864 | nd | nd | 0.26±0.00 | nd | nd | nd | nd | nd |
| 64 | 7-Oxabicyclo[2.2.1]heptane-2,3-dic arboxylic acid | 16.866 | nd | nd | 0.13±0.00 | nd | nd | nd | nd | nd |
| 65 | 2-Octynoic acid | 16.869 | nd | nd | nd | nd | nd | nd | nd | 0.43±0.01 |
| 66 | α-Curcumene | 19.525 | 4.47±0.07a | 1.09±0.03e | 1.22±0.03d | 1.67±0.03c | 0.87±0.01f | nd | 0.42±0.01f | 2.36±0.04b |
| 67 | 4-Aminonicotinonitrile | 19.851 | nd | nd | 0.45±0.00 | nd | nd | nd | nd | nd |
| 68 | 3-Methyl-4-(2,5-xylyl)butyric acid | 19.857 | nd | nd | nd | 0.45±0.00 | nd | nd | nd | nd |
| 69 | Cedr-8-ene | 19.862 | nd | 0.43±0.00 | nd | nd | nd | nd | nd | nd |
| 70 | Dihydrocurcumene | 19.868 | 1.38±0.01a | nd | nd | nd | nd | nd | nd | 0.26±0.00b |
| 71 | Tetradecamethylcycloheptasiloxane | 19.971 | nd | nd | nd | 0.40±0.00 | nd | nd | nd | nd |
| 72 | β-Bisabolene | 20.229 | 0.87±0.01a | nd | 0.27±0.00d | 0.35±0.00c | nd | nd | nd | 0.41±0.01b |
| 73 | β-Sesquiphellandrene | 20.681 | 2.06±0.04a | 0.46±0.01d | 0.37±0.01f | 0.64±0.01b | 0.42±0.00e | nd | nd | 0.56±0.00c |
| 74 | Ambrettolide | 32.371 | nd | nd | nd | 0.11±0.00 | nd | nd | nd | nd |
| 75 | Civetone | 32.536 | nd | nd | nd | 0.09±0.00 | nd | nd | nd | nd |
| All values are presented as the mean ± standard deviation of triplicate determination. Means with different letters within a row are significantly different between sample for the same index (p < 0.05); R.T.: Retention time; nd: not detected. | | | | | | | | | | |

**Fig. S1. The manufacturing process of household and commercial *doenjang*.**

**Fig. S2. Phylogenetic placement of 16S rRNA gene sequences from bacteria of household *doenjang*.** Numbers above each node are confidence levels (%) generated from 1000 bootstrap trees. The scale bar is in fixed nucleotide substations per sequence position.

**Fig. S3. Phylogenetic placement of 16S rRNA gene sequences from bacteria of commercial *doenjang*.** Numbers above each node are confidence levels (%) generated from 1000 bootstrap trees. The scale bar is in fixed nucleotide substations per sequence position.

**Fig. S4. Phylogenetic placement of 26S rRNA gene sequences from yeast of household *doenjang*.** Numbers above each node are confidence levels (%) generated from 1000 bootstrap trees. The scale bar is in fixed nucleotide substations per sequence position.

**Fig. S5. Phylogenetic placement of 26S rRNA gene sequences from yeast of commercial *doenjang*.** Numbers above each node are confidence levels (%) generated from 1000 bootstrap trees. The scale bar is in fixed nucleotide substations per sequence position.

**Figure S1**


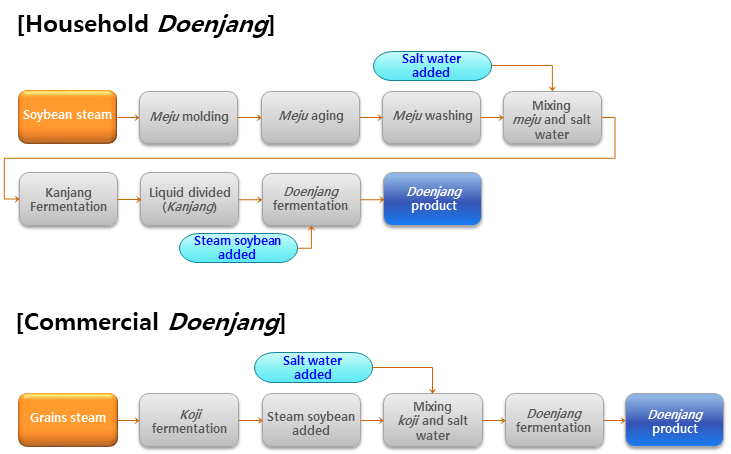


**Figure S2**


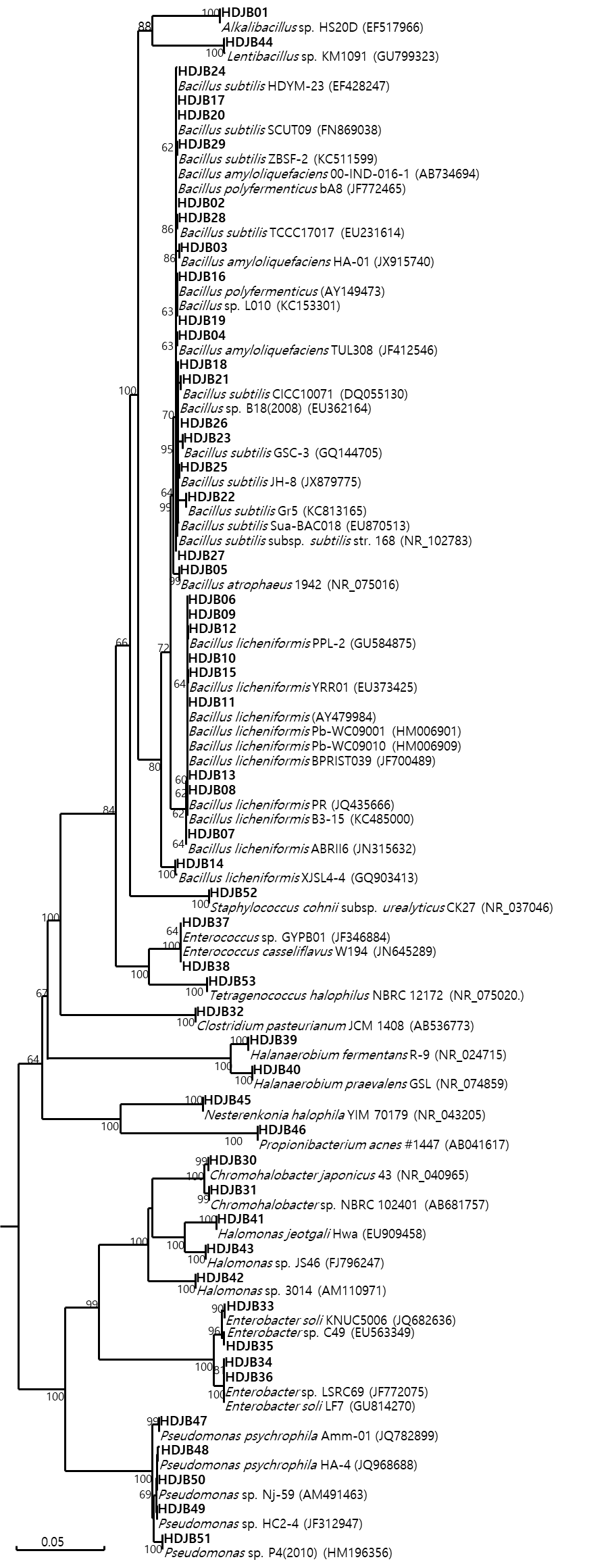


**Figure S3**

**
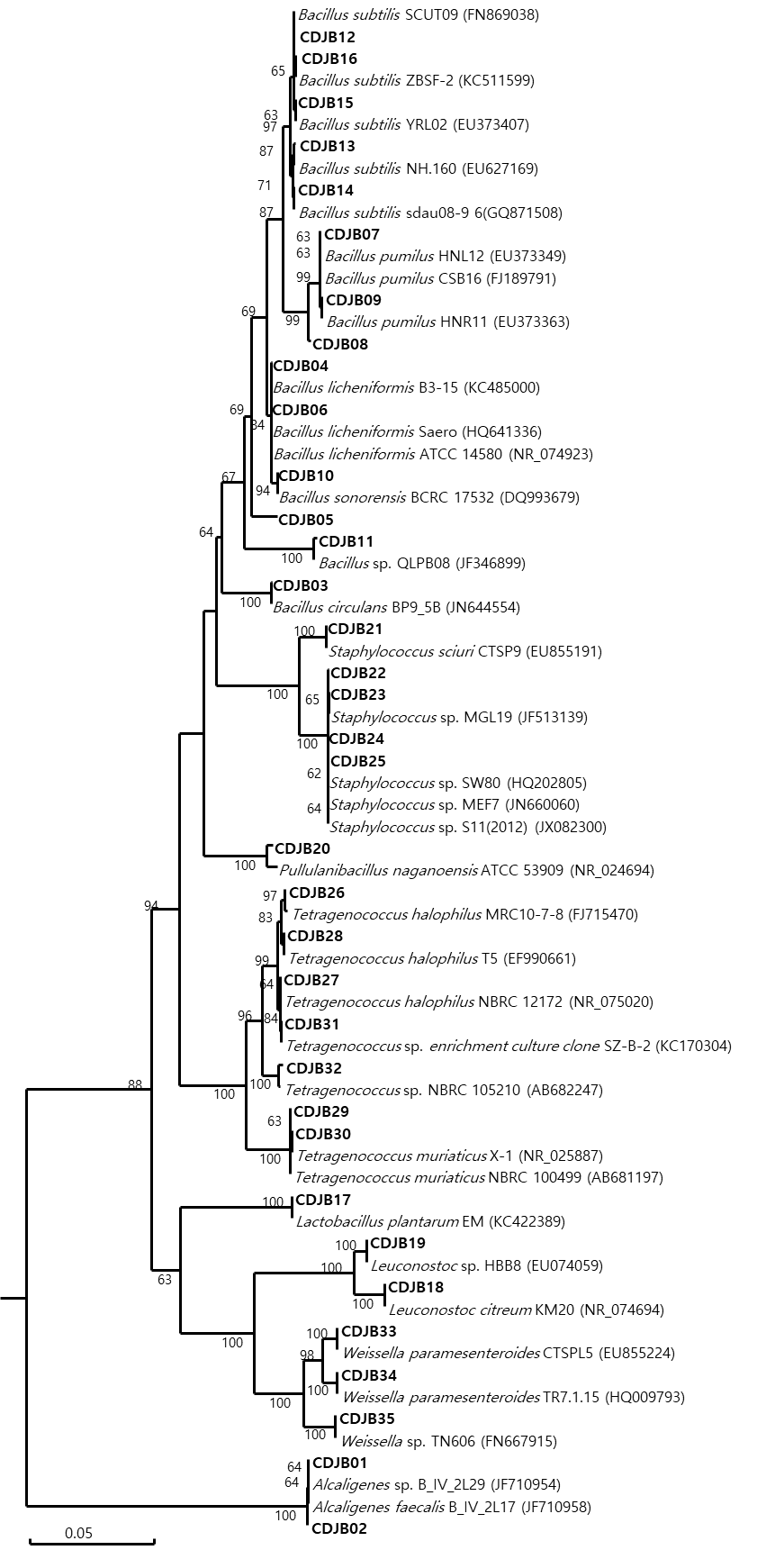
**

**Figure S4**

**
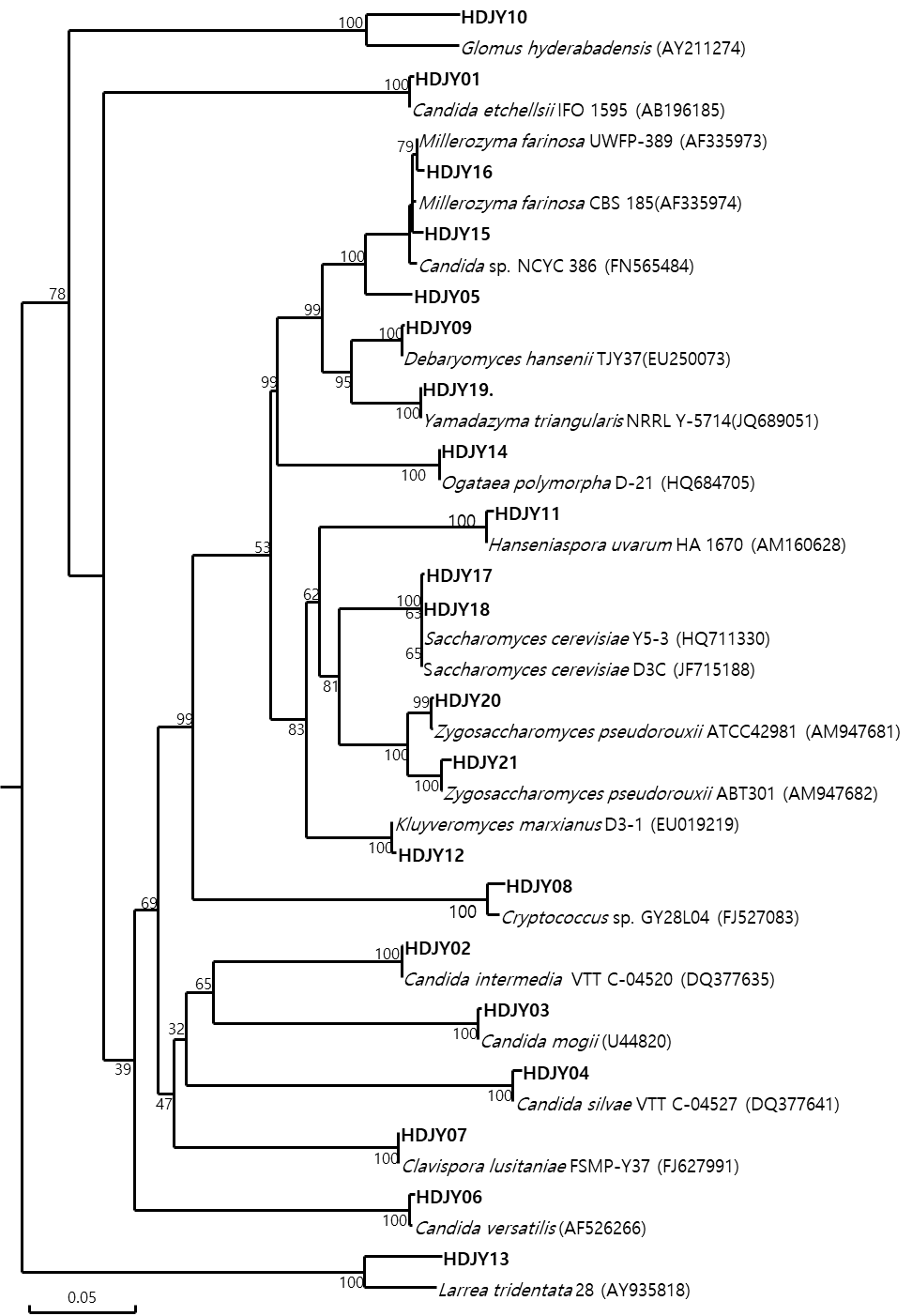
**

**Figure S5**


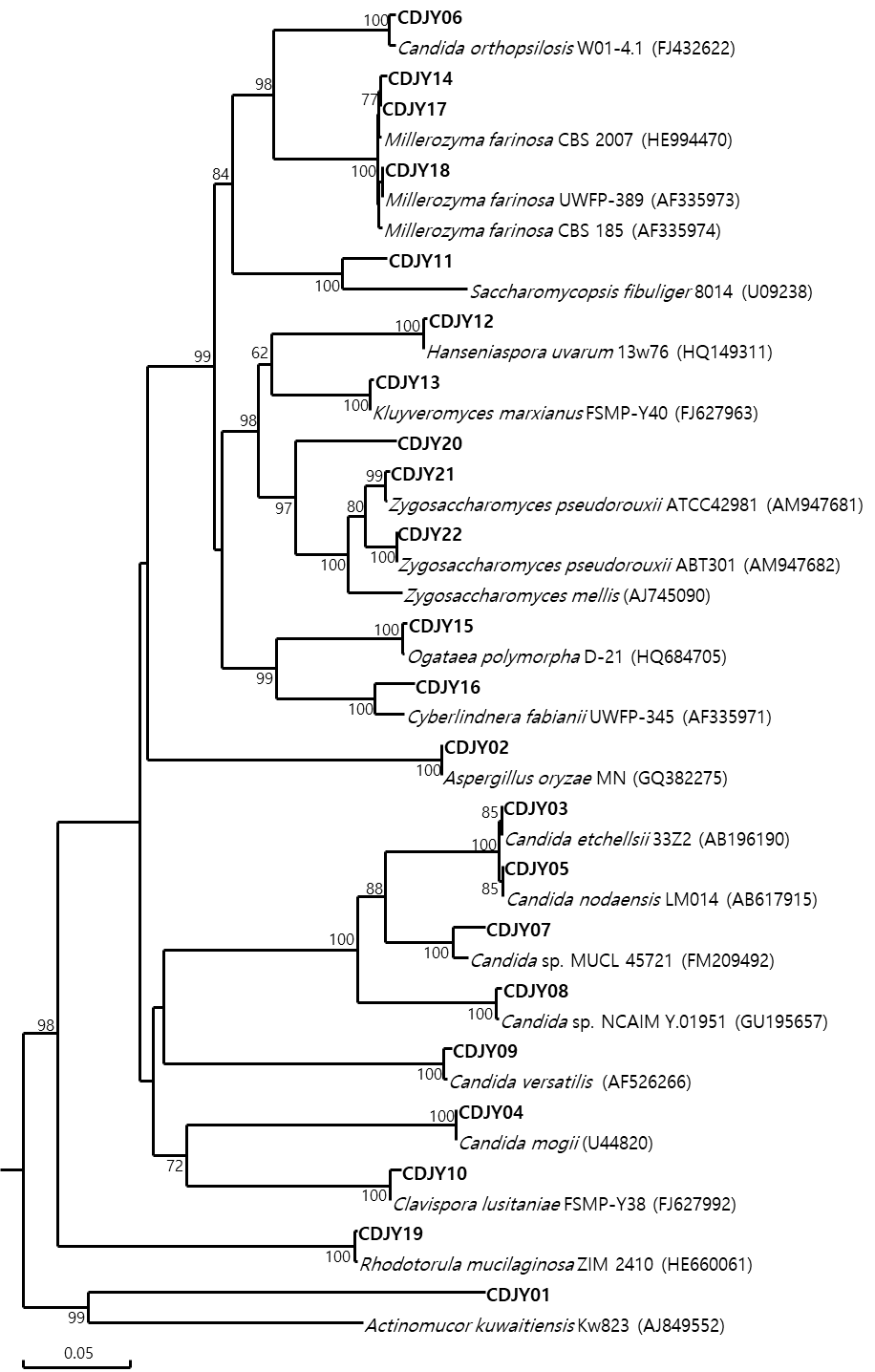

Supplement: Supplementary data 1 [file mmc1.docx]
